# A relative energy gradient (REG) study of the planar and perpendicular torsional energy barriers in biphenyl

**DOI:** 10.1007/s00214-018-2383-0

**Published:** 2018-12-10

**Authors:** Paul L. A. Popelier, Peter I. Maxwell, Joseph C. R. Thacker, Ibon Alkorta

**Affiliations:** 1 Manchester Institute of Biotechnology (MIB), 131 Princess Street, Manchester, M1 7DN UK; 2 School of Chemistry, University of Manchester, Oxford Road, Manchester, M13 9PL UK; 3 Instituto de Química Médica (IQM-CSIC), Juan de la Cierva, 3, 28006 Madrid, Spain

**Keywords:** Quantum chemical topology, QTAIM, IQA, Relative energy gradient (REG), Biphenyl, Energy barrier

## Abstract

Biphenyl is a prototype molecule, the study of which is important for a proper understanding of stereo-electronic effects. In the gas phase it has an equilibrium central torsion angle of ~ 45° and shows both a planar (0°) and a perpendicular (90°) torsional energy barrier. The latter is analysed for the first time. We use the newly proposed REG method, which is an *exhaustive* procedure that *automatically* ranks atomic energy contributions according to their importance in explaining the energy profile of a total system. Here, the REG method operates on energy contributions computed by the interacting quantum atoms method. This method is minimal in architecture and provides a crisp picture of well-defined and well-separated electrostatic, steric and exchange (covalent) energies at atomistic level. It is shown that the bond critical point occurring between the *ortho*-hydrogens in the planar geometry has been wrongly interpreted as a sign of repulsive interaction. A convenient metaphor of analysing football matches is introduced to clarify the role of a REG analysis.

**Electronic supplementary material:**

The online version of this article (10.1007/s00214-018-2383-0) contains supplementary material, which is available to authorized users.

## Introduction

Quantum chemistry has developed into a powerful first principles science, able to make predictions regarding the energy and geometry of molecular systems, independent of experiment. While using less and less computing time, sophisticated algorithms deliver increasingly accurate chemical information. However, quantum chemistry is less conclusive in settling questions on the interpretation of observed phenomena such as torsional energy barriers, whether obtained by computation or experiment.

Biphenyl is a prototype molecule, the study of which is important for a proper understanding of stereo-electronic effects. This simple molecule is of significant interest as a molecular core for many current compounds currently under proposal for clinical trials [1]. This is due to its ability to stereo-control the formation of a drug according to substituent groups and their position in the phenyl ring. As a core it features in 2.6% of all Cambridge Structural Database (CSD) structures and 0.2% of all Protein Data Bank (PDB) structures. In the CSD it is the most frequently observed core and is the seventh most frequent in the PDB.

In 2008, Johansson and Olsen built on previous computational studies [2–5] of biphenyl to carry out the highest level ab initio calculations to date, which attempted to reconcile the computational and experimental torsional energy barriers and equilibrium angle of biphenyl in the gas phase [6]. Their best estimate for the central torsion angle at equilibrium was 45.8°, which lies just outside the experimental interval [7] of 44.4 ± 1.2°, already established in 1985. The energy profile of biphenyl as a function of its central torsion angle has two energy barriers: planar (torsion angle 0°) and perpendicular (90°). The best calculated values to date [6] for the planar and perpendicular torsional barriers are 8.0 kJ mol^−1^ and 8.3 kJ mol^−1^, respectively. Experimentally determined torsional barriers [8] appear to have settled in 1985 on 6.0 ± 2.1 kJ mol^−1^ and 6.5 ± 2.0 kJ mol^−1^ for the 0° and 90° barriers, respectively. Again, the best computed values are near the edge of experimental uncertainty interval. In spite of this encouraging near-agreement, the *interpretation* of these torsional barriers, in terms of back-of-the-envelope chemical insight, has remained controversial.

The reason for this continuing discussion is the same as that fuelling the debate on the origin of the torsional barrier in ethane [9, 10], which is that experiment does not falsify conflicting and coexisting interpretations. This is why a way forward [11] is perhaps Occam’s razor, which supports the interpretation with the smallest number of assumptions. One approach that offers such a minimal interpretation is quantum chemical topology (QCT) [12–15], which is parameter-free, orbital-free, and reference-state-free (all explained in Sect. 2.1). This is the approach we will follow in the current work, particularly the interacting quantum atoms (IQA) methodology [16]. IQA generates both intra-atomic and interatomic energies, thereby providing highly localised information, which lends itself to chemical insight. However, the challenge is that the number of IQA energy contributions quickly becomes overwhelming, rendering a manual analysis tedious or even impossible. How can this challenge be overcome?

The newly proposed relative energy gradient (REG) [17] method comes to the rescue by operating, in a systematic, exhaustive and automatic way, on all IQA energy contributions in biphenyl. The REG method is able to *compute* which *part* of a molecule (or molecular complex) behaves similarly to the *whole* molecule in terms of its total energy profile. The REG method thus answers a fundamental question of chemistry: *how to ascribe the cause of a chemical phenomenon occurring in a given molecule to a part of that molecule*. In this case study, this phenomenon is the planar rotation barrier in biphenyl (the other barrier being the perpendicular one), and its potential cause could be a steric clash between the two *ortho*-hydrogens. The REG method finds this subsystem by minimal assumption and by automatic action on an *exhaustive* set of atomistic energy contributions (of all quantum mechanical types as explained in Sect. 2.2).

The REG method is general and able to explain the gauche effect [18], extract the arrow-pushing scheme (i.e. the reaction mechanism) of an enzymatic reaction (peptide hydrolysis in the HIV-1 protease active site) [19] from more than 17,000 individual IQA energy contributions, and elucidate halogen-alkane nucleophilic substitution $({\rm S}_{{\rm N}^2})$ reactions [20]. A further case study where the REG method is bearing fruit (work to be published) is that of the so-called secondary interaction hypothesis [21]. Here the question is whether one can explain, or even predict, the variation in stability between van der Waals complexes solely based on interactions between their molecular constituents. It has been known for a long time that simply counting hydrogen bonds is not satisfactory. It turns out that the subsystem that explains the total system consists of more atoms than just those involved in the hydrogen bonds.

Returning to biphenyl, its planar energy barrier, which is one of two torsional energy barriers, has been investigated “topologically” and “non-topologically”. An early study [22] of the former type is that of Cioslowski et al. who interpreted the so-called bond critical point between the two bay (i.e. ortho-) hydrogens as a signature of a repulsive H…H interaction. This repulsion is then interpreted as the cause of the planar energy barrier. It is important to note, already at this point, that the current article is not a study of the meaning of the BCP, nor is it triggered by it. Instead, we focus on the atomic energies that IQA provides, which are physical quantities that change smoothly with varying molecular geometry. As such, they do not suffer from a sudden appearance or disappearance of a BCP, nor of potentially erroneous interpretations of what a BCP expresses in energetic terms, be it attraction or repulsion. The IQA energy quantities speak for themselves and do not need a projection of one’s own chemical intuition onto them.

More than a decade later, in 2003, Matta et al. [23] opposed this interpretation of a H_ortho_…H_ortho_ BCP as a signature of repulsive interaction. In their extensive study on hydrocarbons, including polyaromatic and saturated compounds, they introduced hydrogen–hydrogen bonding as a universal phenomenon. They showed that this type of bonding is marked by the presence of a BCP, as a general class of *stabilising* interactions in molecules and crystals. Thus, biphenyl is just another system that contains such an interaction. A few years later, in 2006, Solà and Bickelhaupt refuted [24] the existence of hydrogen–hydrogen bonding in planar biphenyl and confirmed the classical view of steric repulsion between *ortho*-hydrogens. This view was then immediately rebutted [25] by Bader in 2006 and again quickly counter-rebutted [26] by Solà and Bickelhaupt. Subsequently, in 2007, Hernández-Trujillo and Matta revisited the H…H bonding in biphenyl and strengthened their evidence for the interaction between the *ortho*-hydrogens being *locally* stabilising. Using the electrostatic potential at the position of the nuclei, Pacios and Gómez came to the same conclusion [27]. The former authors pointed out that the lengthening of the central C–C bond (joining the two phenyl rings) results in a net destabilisation exceeding the stabilisation of the *ortho*-hydrogens. In other words, the torsional barrier arises through a balancing act between stabilising and destabilising contributions. An important question that then asks itself is: How can one be sure that no energy contributions have been overlooked in this balancing act, and how do they all act together? The REG method offers a general, robust and automatable procedure that can tease out the relevant energy terms, both by locale and by type (steric, electrostatic and exchange)?

We note that up to that point in time, in 2007, a full IQA partitioning of biphenyl was computationally as good as intractable, due to the rather large atomic integration errors and the enormous computing time required. This is why all topological energy considerations had been carried out using the original virial-based atomic energy (calculated from the kinetic energy density over the volume of a single atom). However, in 2007, it was just about possible [28] to calculate an IQA interaction energy between the two *ortho*-hydrogens at the planar and “twisted” equilibrium geometry. That study yielded early evidence that the *ortho*-hydrogens fitted an interaction energy image that cannot be easily distinguished from that found in a (weak) covalent link.

Several years later, in 2014, Eskandari and Van Alsenoy performed [29] a full IQA energy partitioning on biphenyl, complemented with an alternative energy partitioning method called “fractional occupation iterative Hirshfeld”. Both methods confirmed a net attraction between the hydrogens, which is maximised at the planar geometry. The authors used a B3LYP wave function in conjunction with the atomic integration program AIMAll before it had been extended to return IQA energy contributions that add up [30] to the original molecular energy. For a robust implementation of B3LYP using the IQA methodology, it is necessary to explicitly code the functional inside AIMAll. It is known [31] that if this is not done then the IQA energies correlate poorly with the situation where it is done, except for hydrogen atoms. Furthermore, there is an alternative way [32] of taking the explicit functional into account within the IQA framework, which is not implemented in AIMAll. In order to switch off these ambiguities one can work with Hartree–Fock wave functions, as done in this study and also in the knowledge that this level of theory suffices to capture the nature of the torsional barrier.

Most recently, between 2015 and 2018, Jenkins and Kirk et al. [33–36] applied their topological stress tensor and vector-based perspective, as well as the total local energy density, to shed light on the internal connections and stability of bonding patterns in biphenyl and substituted biphenyls. Finally, in 2018, Jara-Cortés and Hernández-Trujillo used IQA to investigate [37] conjugated hydrocarbons including biphenyl. They confirmed that the H…H bonding found in some of the aromatic molecules studied was attractive, according to the stabilising exchange interaction between the bonded H atoms. They claimed that the energy barrier in biphenyl is mainly a consequence of the global decrease of the C–C favourable interactions.

## Methods and theoretical background

### The qualitative nature of quantum chemical topology (QCT)

**Fig. 1:**
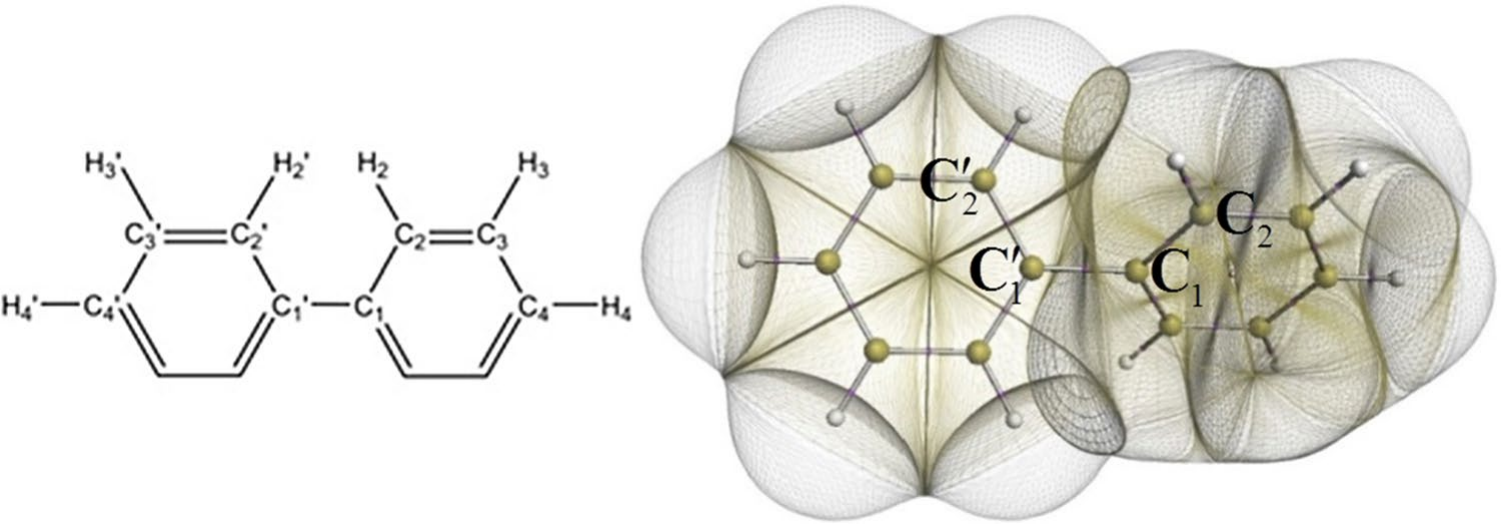
Quantum topological representation [40] of biphenyl at equilibrium. The non-overlapping topological atoms are bounded by interatomic surfaces at the inside of biphenyl and by a constant electron density envelope of 0.001 a.u. at the outside. This picture was generated by the in-house code IRIS [41]. The central torsion angle (*τ* = C_2_′ C_1_′ C_1_ C_2_) controls the geometrical changes in biphenyl where *τ* = 0° and *τ* = 90° correspond to the planar and perpendicular energy barriers, respectively. The labels are part of a scheme that is shown in full later on and used throughout this article. The position of atoms C_5_, H_5_, C_6_ and H_6_ can be easily deduced from the order of the numbering

This section aims at an audience that is perhaps less familiar with a topologically based energy partitioning than with the older “non-topological” energy partitioning schemes, as critically reviewed recently [38]. We start with QCT’s first hallmark: parameter-free atomic partitioning. QCT defines an atom in a molecule (or in any piece of matter such as a molecular cluster or crystal) starting from the electron density *ρ*. The key to achieve this atomic partitioning [39] is simply the gradient of *ρ*, which traces paths of steepest ascent in *ρ*. The vast majority of these paths terminate at a nucleus, and the subspace that such a bundle of paths occupies constitutes the (topological) atom. Figure 1 shows the QCT partitioning of the molecular electron density at the equilibrium geometry of biphenyl. No parameter was used to obtain these atoms.

It is clear that there are no gaps between the atoms and that they do not overlap. Within the interior of the molecule the atoms are separated by the so-called interatomic surfaces [42], while at the outside they are capped by a three-dimensional envelope of constant electron density, just for visual purposes. However, in the condensed matter state, topological atoms are completely bounded by interatomic surfaces. Finally, each point in three-dimensional space (within the molecular envelope) is assigned to a topological atom. This means that each bit of electron density belongs to an atom because no portions of space are unassigned [43]. Consequently, each portion of space adds to the total molecular energy an energy contribution that can be associated with an atom. This assertion follows from the mathematical link (through volume integrals) between energy and electron density.

We now explain the second hallmark of QCT: how and why topological atoms are defined in an orbital-free manner. The electron density is an attractive starting point for an atomic partitioning because the electron density can be obtained in very different ways. Three familiar classes of methods exist: orbital methods, grid-based methods and diffraction experiments. In this work we follow the first class and obtain wave functions expanded in terms of Gaussian primitive functions. A partitioning decision in real space does not suffer from possible instabilities arising in a partitioning in the so-called Hilbert space of basis functions (e.g. Gaussians). However, the most important feature of the topological atom in the current context is that the kinetic energy of a topological atom is well defined [44, 45], within the Laplacian family [46] of local kinetic energies. In other words, arbitrary subspaces have ill-defined kinetic energies. In the original formulation of QCT, the topological atom has its own virial theorem [47], which is a relationship between kinetic and potential energy. As a result, the potential energy of an atom could be obtained from its kinetic energy alone. The condition for this scheme to work was that a molecule had to be at equilibrium, i.e. all forces operating on the nuclei should vanish. However, in 2001, it became possible [48, 49] to calculate routinely the potential energy of an atom residing within a *non*-*equilibrium* molecular geometry. This advance also enabled the calculation of the Coulomb-like potential energy between atoms, again for any given molecular geometry. With the additional calculation of interatomic exchange energy [50] the analysis of torsional barriers became possible [51] in terms of a complete topological energy partitioning [52].

Building on the 2001 approach, Blanco et al. [16] proposed a new algorithm in 2005 for the computation of the three primary types of energy contribution that underpin the current work on biphenyl. This led to a series of papers under the umbrella of what these authors called *interacting quantum atoms* (IQA). They successfully used IQA to reformulate hydrogen bonding [53], stereo-electronic effects [54] and bonding in first-row diatomic molecules [55]. The IQA method has also been fruitfully used to gain meaningful insight into a growing number of chemical systems ranging from proton transfer reactions [56], intramolecular bond paths between electronegative atoms [57], hydrogen–hydrogen interactions with respect to the torsional barrier in biphenyl [29] over CO_2_ trapping by adduct formation [58], connecting Buckingham-type potentials to atom–atom repulsion [59] and the diastereoselective allylation of aldehydes [60], to name a few. We note that an extensive comparison [61] between IQA and alternative non-QCT methods showed that IQA (and hence QCT) gives a less distorted image of chemical phenomena, leading to smaller deformation and interaction energies, thus better preserving the atomic identity from the energetic point of view.

The third and final QCT hallmark is its *reference*-*state*-*free* nature. Indeed, QCT analyses a single wave function to gain insight into the system at hand, without ever invoking a reference state. One example of a reference state is a wave function that has not been anti-symmetrised and thereby violates the Pauli principle. Another example is a promolecule, which is a simple superposition of free atoms, lacking any hybridisation. Such states are typically artificial in that they cannot be realised in nature and may require further parameters to be set. Secondly, at the level of the electron density itself, the gradient at the heart of QCT partitioning does not invoke a reference electron density either. In fact, the molecular electron density acts as its own reference because the gradient is essentially an *internal* difference. In other words, the pattern that the gradient creates follows from an inspection of the electron density relative to itself.

### Topological energy partitioning

This topic has been reviewed many times, which is why we reiterate only the essence here. The first-order reduced density matrix *ρ*_1_ and the second-order reduced density matrix *ρ*_2_ are the basic quantities one needs to define the three primary energy contributions: the intra-atomic energy *E*_intra_, the interatomic electrostatic energy (typically denoted as “classical” or “cl”) *V*_cl_ and the interatomic exchange energy *V*_x_. In order to align our text with the IQA literature, including the original paper [16], we will use the peculiar (and improvable) shorthand notation of the various IQA energy contributions, except *E*_net_, which is better called *E*_intra_. We also note that the original definition of *ρ*_2_ by Löwdin [62] introduces a more logical factor of ½ in front of the integral. However, for some reason, McWeeny drops [63] this factor of ½, a convention that the authors of IQA adopted.

On a technical note it is also possible to partition electron correlation energy within the IQA framework, be it originating from a DFT wave function [30, 64], a MP2 (or MP3 or MP4) wave function [65], or a coupled cluster wave function [66]. However, for reasons explained in Sect. 2.3, the current study does not involve any electron correlation. Indeed, the torsional barriers in biphenyl can be explained by the interplay of these three primary energy contributions.

The intra-atomic energy collects all energy terms that concern a single atom A, that is, the kinetic contribution *T*_A_, the nuclear–electron attraction and the electron–electron repulsion within *A*,

1 $$E_{\text{intra}}^{\rm A} = T_{A} + V_{\rm ee}^{\rm AA} + V_{\rm en}^{\rm AA}$$

The second primary energy contribution, denoted as $V_{\text{cl}}^{\rm AB}$, is the quantum mechanical equivalent of the classical electrostatic (or Coulomb) interaction between two charged objects, in this case atoms *A* and *B*. In order to calculate $V_{\text{cl}}^{\rm AB}$ one needs also the classical nucleus–nucleus repulsion, $V_{\rm nn}^{\rm AB}$, and the generalisation of the electron–nucleus attraction appearing in Eq. 1, $V_{\rm en}^{\rm AB}$, where now the electrons of atom *A* interact with the nucleus of atom *B* (instead of that of the same atom *A*). The order of subscripts matters because $V_{\rm en}^{\rm AB} \ne V_{\rm ne}^{\rm AB}$, in general. The final contribution needed to complete the definition of $V_{\text{cl}}^{\rm AB}$ is the interaction energy between the electron density (i.e. the purely electronic density, rather than the charge density, which includes the nuclear density) within atom *A* and within atom *B*, denoted as $V_{\text{coul}}^{\rm AB}$. Combining all electrostatic energy contributions leads to

2 $$V_{\text{cl}}^{\rm AB} = V_{\rm coul}^{\rm AB} + V_{\rm en}^{\rm AB} + V_{\rm ne}^{\rm AB} + V_{\rm nn}^{\rm AB}$$

It is clear from this equation that $V_{\text{cl}}^{\rm AB}$ covers all four possibilities: electron–electron, electron–nucleus, nucleus–electron and nucleus–nucleus, but ignores exchange–correlation between electrons. This is why this contribution is denoted as *V*_*cl*_, where the “cl” refers to the “classical” electrostatic interaction, which excludes pure quantum effects. In this work the exchange–correlation contribution is confined to exchange only and is given by $V_{\text{x}}^{{\varOmega_{1} \varOmega_{2} }}$. This important contribution is part of $E_{\text{intra}}^{\rm A}$ (as $V_{\text{x}}^{\rm AA}$) and constitutes the third primary energy distribution, denoted as $V_{\text{x}}^{\rm AB}$. We can then combine $V_{\text{x}}^{\rm AB}$ and $V_{\text{cl}}^{\rm AB}$ into another useful potential energy quantity called $V_{\text{inter}}^{\rm AB}$, which is defined as follows,

3 $$V_{\text{inter}}^{\rm AB} = V_{\text{x}}^{\rm AB} + V_{\text{cl}}^{\rm AB}$$

which describes the complete interaction between two different atoms *A* and *B*. One more useful equation should be added. Equation 4 defines the total IQA energy of a single atom *A*, $E_{\text{IQA}}^{\rm A}$, as the sum of its intra-atomic energy and the full interatomic potential energy involving all atoms, with which atom *A* interacts, other than *A,*

4 $$E_{\rm IQA} = \sum\limits_{A} {E_{\text{IQA}}^{\rm A} } = \sum\limits_{A} {\left( {E_{\text{intra}}^{\rm A} + \frac{1}{2}\sum\limits_{B \ne A} {V_{\text{inter}}^{\rm AB} } } \right)} = \sum\limits_{A} {\left( {E_{\text{intra}}^{\rm A} + V_{\text{inter}}^{\rm A} } \right)}$$

### Computational details

The data set consists of 11 biphenyl configurations in total, including the equilibrium geometry with central torsion angle (*τ* = C_2_′ C_1_′ C_1_ C_2_, Fig. 1) at 47.6°, and 10 geometries fully optimised, except for the central torsion angle, which was parametrically fixed at values between 0° and 90°, with increments of 10°. The Hartree–Fock dihedral value of 47.6° is only 3.2° larger than the experimental [7] value of 44.4°, while the best computational value [6] exceeds the latter by 1.4°. All geometries were optimised, and wave functions were obtained at RHF/6-311++G(d,p) level, using the tight optimisation option within the program GAUSSIAN09 [67] as well as “6D and 10F” (referring to 6 d-type Gaussians and 10 f-type Gaussians). Nine geometries were optimised under *D*_*2*_ symmetry (i.e. 3 mutually orthogonal twofold rotation axes), while *D*_*2h*_ symmetry was used for *τ* = 0° and *D*_*2d*_ symmetry for *τ* = 90°. Out of a total of 22 atoms only 7 atoms are unique, conveniently chosen as the upper right “quadrant” in the numerical labelling scheme of Fig. 1, consisting of C_1_, C_2_, C_3_, C_4_, H_2_, H_3_ and H_4_. Thus, only about a third of atoms (7/22 = 0.32) needs to be monitored and discussed. Using each of the 7 unique atoms as a generator for a subset of atoms, the full set of atoms can be organised in 7 subsets: {**C**_**1**_, C_1_′}, {**C**_**2**_, C_2_′, C_6_, C_6_′}, {**C**_**3**_, C_3_′, C_5_, C_5_′}, {**C**_**4**_, C_4_′}, {**H**_**2**_, H_2_′, H_6_, H_6_′}, {**H**_**3**_, H_3_′, H_5_, H_5_′} and {**H**_**4**_, H_4_′} where the unique generating atom is marked in bold, and the respective multiplicities are 2, 4, 4, 2, 4, 4 and 2, summing up to 22.

Others have confirmed that Hartree–Fock is a viable option to study biphenyl. In his 2002 study [68] on twist angles and torsional energy barriers in biphenyl and substituted biphenyl, Grein compared optimised dihedral angles using the Hartree–Fock, MP2 and B3LYP methods, as well as using 6-31G* and 6-311+G*. He concluded that “a good choice of basis set is more important than the choice of method, and HF/6-311+G* values are overall quite acceptable for the dihedral angles”. The importance of a decent basis set is echoed in the 2005 study [2] by Sancho-García and Cornil, who stated that “the basis set effects become more critical than the correlation effects beyond MP4 or CCSD(T) levels of theory to obtain the most accurate results”.

Secondly, the HF level can be justified on the grounds that exchange is a much larger effect than electron correlation, which also surfaces in the IQA context. For example, earlier work [30] extensively compared B3LYP and HF energies and found that the trends and relative patterns are not qualitatively different (see Fig. 5 for triglycine or Fig. 8 for the alloxan dimer in that publication). The introduction of B3LYP invariably stabilised 1, 4 and higher interactions, showing a consistent energy shift towards increased exchange energy, in terms of absolute value. Also, only C–C and C–H interactions are the 1,2 interactions with greater $V_{\text{x}}^{\rm AB}$ energies using HF orbitals (over Kohn–Sham orbitals). Furthermore, it was shown [16], already in 2005, that correlated wave functions (FCI or CAS) possess $V_{\text{x}}^{\rm AB}$ values that differ by about 5% compared to those from HF wave functions.

The IQA energy contributions, first obtained using the topological energy partitioning program PROMOLDEN [69] (in the summer of 2013), were not adding up accurately enough to recover the original total energy, which is why the alternative program AIMAll [70] was invoked a year later. No symmetry was used in the calculation of the IQA energy contributions. All atomic properties require numerical quadrature in order to complete the integration over the volumes that the topological atoms occupy. This computationally expensive process introduces a numerical error that we monitored in order to guarantee the robustness of the atomic energies essential to our interpretation. One stringent way of checking the quality of the atomic integration is summing all intra- and interatomic energy contributions and measuring the deviation from the original non-partitioned molecular energy. From Table S1 in the Electronic Supplementary Material (ESM) it is clear that the absolute values of the energy deviation are always below 0.5 kJ mol^−1^ for any of the 11 configurations, which is 4% or 9% of the planar or perpendicular torsional energy barrier, respectively. The energy deviation ranges from − 0.48 to + 0.38 kJ mol^−1^, and the mean unsigned error is 0.24 kJ mol^−1^. In summary, the “integration noise” is sufficiently low to draw confident conclusions from the various energy profiles, many of which will be seen to span intervals larger than that of the total energy.

**Fig. 2:**
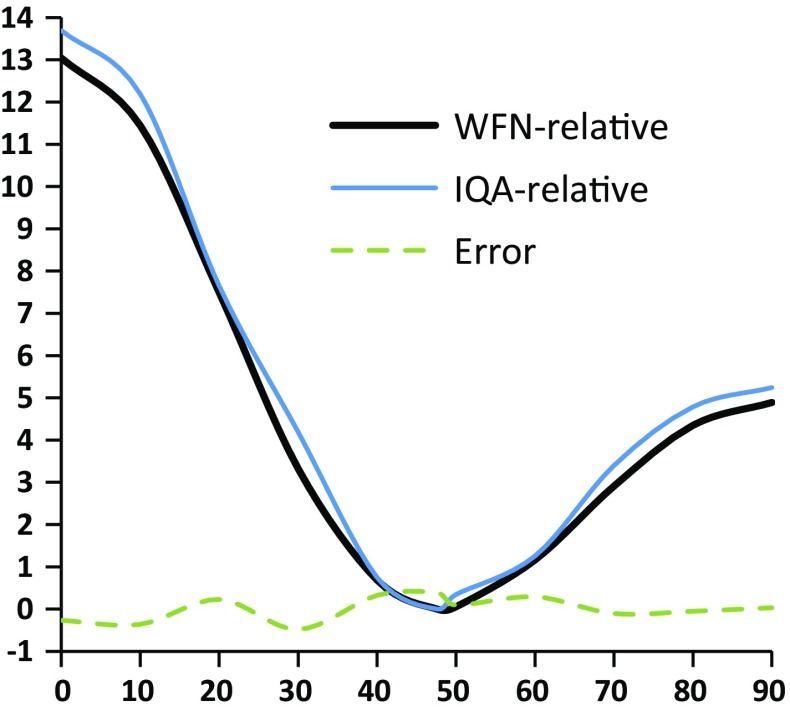
IQA (blue, obtained by summing all IQA energy contributions) and ab initio (black, original wave function (wfn)) molecular energies (in kJ mol^−1^, both relative to the minimum geometry at 47.6°) for biphenyl configurations (geometry-optimised except for the central torsion angle, which was parametrically fixed at values between 0° and 90°, sampled every 10°). The discrepancy (“error”, kJ mol^−1^) between the IQA and original ab initio energy is marked as a green dashed line (corresponding values are in Table S1 of the ESM)

Figure 2 shows the molecular torsional energy profile (plotted in black), with a planar barrier of 13.0 kJ mol^−1^ (left) and a perpendicular barrier of 4.9 kJ mol^−1^ (right). This figure also shows the “energy recovery error” (IQA-summed *versus* original molecular energy) as a green dashed line, illustrating the smallness of the integration noise.

Finally, we point out that the geometric relaxation of the whole molecule, allowed during the torsional rotation, induces changes in internuclear distances and in $V_{\text{inter}}^{\rm AB}$, $V_{\text{x}}^{\rm AB}$ and $V_{\text{cl}}^{\rm AB}$. Table S2 of the ESM monitors the respective ranges (i.e. difference between maximum and minimum values encountered) of these changes. As expected, the CC distance ranges decrease in the sequence of bonds C1–C1′, C1–C2, C2–C3 and C3–C4 as one moves away from the central torsion angle. (A picture with atomic labels is provided in the ESM near Table S2.) Similarly, the C2–H2 distance range is dramatically larger than that of C3–H3 and C4–H4, due to the proximity of C2–H2 to the central torsion angle.

### The relative energy gradient (REG) method

It turns out that a system with only 22 atoms, such as biphenyl, is already generating hundreds of IQA energy contributions. Indeed, if there are *n* atoms then there are *n*(*n* − 1)/2 atom pairs. For each pair (A,B) there are two types of primary interatomic energies $V_{\text{x}}^{\rm AB}$ and $V_{\text{cl}}^{\rm AB}$, while there are *n* intra-atomic energies $E_{\text{intra}}^{\rm A}$. As a result, there are 2[*n*(*n* − 1)/2] + *n* =* n*^2^ primary energy terms making up the total energy of a system with *n* atoms. In particular, biphenyl’s potential energy surface (PES) consists of 22^2^ = 484 energy terms that together govern biphenyl torsional energy barriers. The crucial question is *which subset of terms can be shown, by computation, to be responsible for the energy barriers.* Answering this question, exhaustively and essentially without bias, is a cornerstone of this article. We now explain how this goal is achieved.

The relative energy gradient (REG) method (for full details see Ref. [17]) is designed for systematically ranking the partitioned energies into an order expressing how they contribute, and thus best describe, the behaviour of a molecule’s PES. There are two main aims of the REG method: (1) extracting chemical insight from an energetically partitioned system and (2) determining *subsets* of partitioned energies that best describe how the total energy changes over the PES. The REG method is automated by the in-house program ANANKE and therefore allows for the *exhaustive* analysis of all IQA energy terms in a system. Automation of the REG method is important because the total number of energy contributions ($E_{{\text{int} {\text{ra}}}}^{\rm A}$, $V_{cl}^{\rm AB}$ and $V_{x}^{\rm AB}$) quadratically increases with the number of atoms, rapidly making manual analysis unfeasible.

The REG method is so coined because it compares the gradient of a given energy contribution *E*_*i*_ against the gradient of the total energy, *E*_total_, using linear regression,

5 $$E_{i} (s) = m_{{{\text{REG}},i}} E_{\text{total}} (s) + c_{i}$$

where *s* is a control coordinate, $m_{{{\text{REG}},i}}$ is the REG (from here onwards referred to as REG_*i*_), and *c*_*i*_ is the y-intercept, which has no chemical meaning in this context. The control coordinate is responsible for generating the energy profile; in the current case study, this coordinate is the central torsion angle *τ* = C_2_′ C_1_′ C_1_ C_2_. In the study of a hydrogen bond, however, the control coordinate is typically the distance between the hydrogen nucleus of the donor and the oxygen nucleus of the acceptor, for example. Note that Eq. 5 actually lists an equation for every energy contribution *i*, one that is fitted to the data points that represent the energy profile being analysed. The control coordinate generates these data points, that is, a series of snapshots of biphenyl between *τ* = 0° and *τ* = 90°.

The *REG*_*i*_ can be estimated using ordinary least squares linear regression as shown in Eq. 6,

6 $$m_{{{\text{REG}},i}} = \frac{{\left( {{\mathbf{E}}_{\text{total}}^{\text{translated}} } \right)^{T } \cdot {\mathbf{E}}_{i}^{\text{translated}} }}{{\left( {{\mathbf{E}}_{\text{total}}^{\text{translated}} } \right)^{T } \cdot {\mathbf{E}}_{\text{total}}^{\text{translated}} }}$$

$${\text{where }}\begin{array}{*{20}c} {\left( {{\mathbf{E}}_{i}^{\text{translated}} } \right)^{T } = \left[ {\begin{array}{*{20}c} {E_{i} (s_{1} ) - \bar{E}_{i} } & {E_{i} (s_{2} ) - \bar{E}_{i} } & \cdots & {E_{i} (s_{M} ) - \bar{E}_{i} } \\ \end{array} } \right]^{T } } \\ {\left( {{\mathbf{E}}_{\text{total}}^{\text{translated}} } \right)^{T } = \left[ {\begin{array}{*{20}c} {E_{\text{total}} (s_{1} ) - \bar{E}_{\text{total}} } & {E_{\text{total}} (s_{2} ) - \bar{E}_{\text{total}} } & \cdots & {E_{\text{total}} (s_{M} ) - \bar{E}_{\text{total}} } \\ \end{array} } \right]^{T } } \\ \end{array}$$

and the superscript bar represents an average over all *M* data points, while the energy translation results from the subtraction of the respective averages, and *T* denotes the transposition of a standard column vector into a row vector.

The validity of the linear regression analysis is tested using the Pearson correlation coefficient *R*, one coefficient for each energy contribution *i*. If the magnitude of *R* is close to 1 then the REG value is valid. As *R* moves away from 1 the REG concept deteriorates because then it becomes less and less meaningful to compare the gradients of *E*_*i*_ and *E*_total_ using a linear model. This fact is illustrated in figure 4 of Ref. [17], where the REG method is discussed for the first time and in great detail. Figure 2 in the current article also helps understand why a high *R* value is necessary to have a chemically meaningful REG. Figure 2 shows that the total energy profile consists of two segments: one segment monotonically decreasing from the planar barrier (left, *τ* = 0°) to the energy minimum, and the other segment monotonically increasing from the minimum to the perpendicular barrier (right, *τ* = 90°). The REG method seeks an atomic IQA energy contribution *E*_i_(*τ*) that behaves in the same way as the total energy *E*_total_(*τ*), over a given stretch of the PES, i.e. an interval of the central torsion angle *τ*. Equation 5 is tasked with this search and looks for a (largely) *constant* ratio between *E*_i_(*τ*) and *E*_total_(*τ*) *over a single continuous stretch.* Such a ratio can typically be found (i.e. with *R* near to 1) over one segment only, while another segment will return a *different* ratio. In other words, if one looks for a *single* ratio covering *both* segments then the price to pay is a serious deterioration of the *R* value, to the point of being meaningless. In summary, the REG method operates on one segment at a time and is thereby able to potentially find different *E*_i_(*τ*) contributions, explaining the barrier governing each segment by a different chemical reason.

Next, we explain the semi-quantitative interpretation of the REG values. For convenience we will simply call the segments of the previous paragraph *barriers.* The left segment will be referred to as the *planar barrier*, and the right segment as the *perpendicular barrier*. For clarity, each barrier is bounded by two stationary points (“turning points”) in the one-dimensional energy profile (see Fig. 2). An energy contribution *E*_i_(*τ*) with a *positive* REG_*i*_ value contributes to the barrier in the *same direction* as the total molecular system (i.e. has the same behaviour as the total system over this barrier). In contrast, a *negative* REG_*i*_ term contributes in the opposite direction of the total system over a barrier (i.e. this REG_*i*_ term has the opposite behaviour to the total system over the given barrier). Secondly, the magnitude of a REG_*i*_ value is also important. This magnitude is the absolute value of the ratio of *E*_i_(*τ*) to *E*_total_(*τ*). As a result, a REG_*i*_ value with a magnitude greater than 1 contributes to a given behaviour to a greater degree than the total system does. On the other hand, a REG_*i*_ with an absolute value less than 1 contributes less to the behaviour, over a given barrier, than the total system does. Thus, the larger the magnitude of a REG_*i*_ value, the more important it is in determining the total behaviour of the system.

In summary, a large positive REG_*i*_ values contributes most to the total behaviour of the system, while a large negative REG_*i*_ value contributes most to opposing the total behaviour of the system. Ranking the energies from largest to smallest produces an ordered list of IQA energy terms that directly contribute towards (or against) a given barrier. This list provides a chemically intuitive interpretation for each barrier.

## Results and discussion

### REG analysis: extracting chemical insight allowing for the three different energy types

The REG analysis is the key contribution of the current article to the literature on biphenyl rotations barrier, which is why we report it first. However, the ESM reports extensively, by means of figures, tables and full text, data and observations that support the conclusions of the REG analysis.

**Fig. 3:**
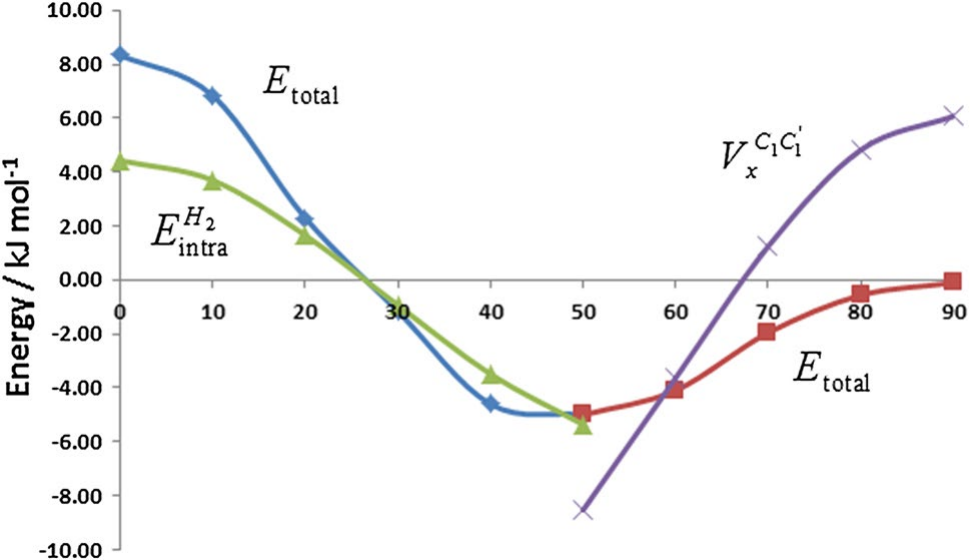
The planar (left, blue lozenges) and perpendicular energy barriers (right, red squares) as a function of the central torsion angle. Each barrier is accompanied by the energy profile of the IQA term with the largest REG_i_ value: $E_{\text{intra}}^{{{\text{H}}_{ 2} }}$ (left, green triangles) and $V_{x}^{{{\rm C}_{1} {\rm C}_{1}^{'} }}$ (right, purple crosses). Note that, in agreement with Eq. 6, the three energy profiles ($E_{\text{total}}^{{}}$,$E_{\text{intra}}^{{{\text{H}}_{ 2} }}$ and $V_{x}^{{{\rm C}_{1} {\rm C}_{1}^{'} }}$, where the latter two are of the type $E_{i}^{{}}$) are translated by their respective average energy (averaged over all torsion angles). To see this downward translation one should compare the blue profile with that in Fig. 2

We start with full resolution: the overall energy is partitioned both by atom and by energy type (electrostatic, self and exchange). In order to gain an immediate appreciation of the significance assigned to REG_i_ values, it is instructive to understand the REG analysis visually before inspecting the table of REG_i_ values that it generates. Figure 3 shows the two IQA energy terms with the largest positive REG values to both barriers (i.e. the total energy profile covering the planar and perpendicular barriers): $E_{\text{intra}}^{{{\text{H}}_{ 2} }}$ for the planar barrier and $V_{x}^{{{\rm C}_{1} {\rm C}_{1}^{'} }}$ for the perpendicular barrier. The former has a REG_i_ value of 2.78, while the latter adopts a value of 2.94. Intuitively, these two energy contributions express most how their respective barriers behave: their large gradients lead the dynamic character of the barrier, that is, these individual energy terms push hardest in creating their respective barrier. Thus, they can be seen as the overriding chemical cause for the barrier, as we will see below.

Table 1 shows the REG_i_ values with large absolute values for both the planar and the perpendicular barrier, ranked from the highest (positive) value to the lowest (negative) value. We discuss the energy contributions with the absolute largest REG_i_ values first. In order to create a flowing chemical narrative 5 important energy terms (bold in Table 1) will be discussed in the following order: *E*_intra_(H_2_), *V*_x_(C_1_–C_1_′), *E*_intra_(C_1_), *V*_x_ (H_2_–H_2_′) and *V*_x_ (C_1_–C_2_). This order means that the respective energy terms helping the construction of each of the two barriers will be discussed first, and then the terms opposing either barrier.

**Table 1:** The largest REG_i_ values (in absolute value) corresponding to the full IQA partitioning calculated by the program ANANKE

Planar barrier		Perpendicular barrier	
Energy term	REG	Energy term	REG
***E***_**intra**_ **(H**_**2**_ **)**	2.78	***V***_***x***_ **(C**_**1**_–**C**_**1**_**′)**	2.94
*V*_*x*_ (C_1_–C_2_)	1.31	*E*_intra_(C_1_)	2.72
*E*_intra_(C_2_)	1.26	*V*_*x*_ (C_2_–H_2_′)	2.61
*E*_intra_(C_3_)	0.55	*V*_*x*_ (C_2_–C_3_)	2.18
*V*_*x*_ (C_1_–H_2_′)	0.54	*V*_*x*_ (H_2_–H_2_′)	1.00
*V*_*x*_ (C_1_–C_2_′)	0.46	*V*_*x*_ (C_2_–C_2_′)	0.83
V_cl_ (C_2_–H_2_)	− 0.48	*E*_intra_(C_2_)	− 0.80
*V*_*x*_ (C_2_–H_2_′)	− 0.55	*V*_*x*_ (C_1_–C_2_′)	− 0.81
*V*_*x*_ (C_2_–C_3_)	− 0.70	*V*_cl_ (C_1_–C_1_′)	− 0.82
***V***_***x***_ **(H**_**2**_–**H**_**2**_**′)**	− 1.74	E_intra_(H_2_)	− 1.67
***E***_**intra**_ **(C**_**1**_ **)**	− 2.26	***V***_***x***_ **(C**_**1**_–**C**_**2**_**)**	− 4.59

Positive REG_i_ values assist the respective energy barrier, while negative REG_i_ values work against the barrier. The five bold values are discussed first and in the following order: *E*_intra_ (H_2_), *V*_x_(C_1_–C_1_′), *E*_intra_ (C_1_), *V*_x_ (H_2_–H_2_′) and *V*_x_ (C_1_–C_2_). Atomic multiplicity is taken into account

**Fig. 4:**
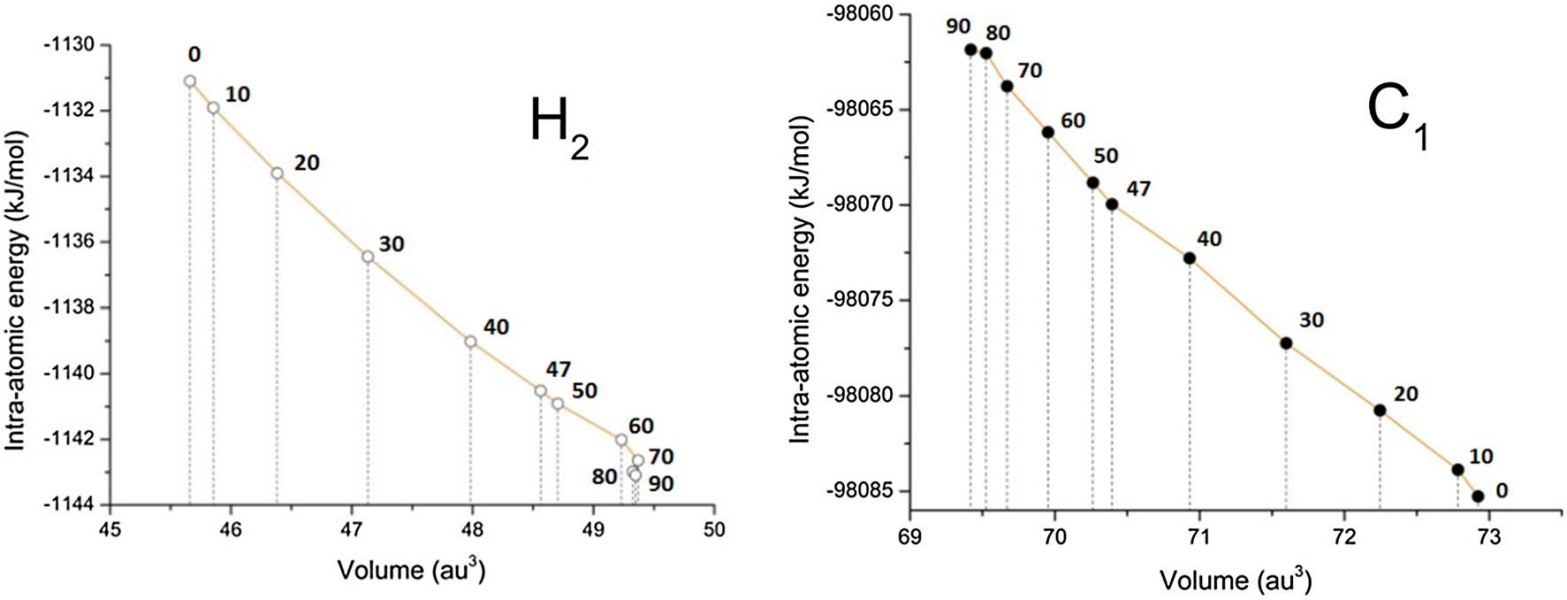
The atomic intra-atomic energy as a function of the corresponding atomic volume (for the 0.001 a.u. constant electron density envelope) of the two atoms (H_2_ and C_1_) that change most dramatically with variation of the central torsion angle (*τ* = C_2_′ C_1_′ C_1_ C_2_ marked along the profiles, in degrees)

Table 1 confirms that $E_{\text{intra}}^{{{\text{H}}_{ 2} }}$ is the one energy contribution (REG_*i*_ = 2.78) that behaves most like the planar barrier. We showed in previous work [59] that $E_{\text{intra}}^{{}}$ quantifies steric interaction by mutual atomic deformation. Thus, chemically this means that a steric clash between *ortho*-hydrogens is the main cause for the planar barrier. *The finding is compatible with the standard textbook interpretation*. As a quick aside we keep in mind that the quantity that mostly influences $E_{\text{intra}}^{{}}$ is charge transfer. Indeed, adding or extracting electrons from a topological atom has a large impact on $E_{\text{intra}}^{{}}$. However, this effect is not important for H_2_ (or later C_1_) because of the minimal charge transfer in biphenyl, which amounts to hydrogens being only slightly negative (−0.01e rounded off) and carbons slightly positive (+ 0.01e). Continuing with the main argument, we can say that any energy contribution that helps the construction of a barrier is mandatorily destabilising, by deduction. Thus, in going from the equilibrium geometry to the planar geometry, H_2_ becomes increasingly less stable, that is, its own energy increases. This effect is related to a decrease in the atomic volume of H_2_. Figure 4 (left panel) shows $E_{\text{intra}}^{{{\text{H}}_{ 2} }}$ as a function of H_2_′s volume. Indeed, this atom shrinks (to less than 46 a.u.) as biphenyl becomes more and more planar. This figure also shows that, near *τ* = 90°, H_2_ attains its largest volume along the rotation trajectory. In this most expanded or relaxed shape, H_2_ is then also the most stable at this perpendicular conformation. We keep this relationship in mind for the discussion below, on $V_{x}^{{{\rm H}_{2} {\rm H}_{2}^{'} }}$.

The second of the most important energy terms is $V_{x}^{{{\rm C}_{1} {\rm C}_{1}^{'} }}$, now looking at the perpendicular barrier. Table 1 confirms that $V_{x}^{{{\rm C}_{1} {\rm C}_{1}^{'} }}$ behaves most like this barrier. This energy contribution helps construct this barrier, and thus its energy increases (i.e. less negative or destabilising) while approaching the perpendicular conformation. $V_{x}^{{{\rm C}_{1} {\rm C}_{1}^{'} }}$ expresses the covalent energy of the central CC bond, and when its weakening is the main cause for the perpendicular barrier. Note as well the ${\rm C}_{1} {\rm C}^{\prime}_{1}$ bond length is increasing, by 0.006 Å, from its lowest value of 1.492 Å (at the equilibrium position *τ* = 47.6°) to 1.498 Å (for *τ* = 90°). A second way of linking the behaviour of $V_{x}^{{{\rm C}_{1} {\rm C}_{1}^{'} }}$ to familiar grounds involves conjugation. Conjugation is decreased between the two phenyl rings when going from the energy minimum (47.6°) to the perpendicular geometry (90°). In overall summary, each barrier is explained in a chemically simple way, yet compatible with an unbiased automated procedure operating on minimally defined energy contributions.

We now discuss the largest energy terms (in absolute value) that work *against* their respective energy barriers, starting with the third term in the list. The planar barrier is opposed most by $E_{\text{intra}}^{{{\rm C}_{1} }}$ because this energy contribution has the most negative REG_i_ value, namely − 2.26. Any energy contribution that opposes a barrier is mandatorily stabilising, by deduction. Indeed, if the contribution were destabilising it would aid the construction of the barrier. Thus, in going from the equilibrium geometry to the planar geometry, C_1_ becomes increasingly stable, that is, its own energy decreases. This effect is related to an increase in the atomic volume of C_1_. Figure 4 (right panel) shows $E_{\text{intra}}^{{{\rm C}_{1} }}$ as a function of C_1_′s volume. Indeed, this atom swells (to nearly 73 a.u.) as biphenyl becomes more and more planar. This figure also shows that, at *τ* = 90°, C_1_ has the smallest volume. In this most shrunk shape, C_1_ is also the least stable along the rotation trajectory, at this perpendicular conformation. For reference, all volumes of Fig. 4 (and those at the 0.002 and 0.0004 a.u. constant electron density contour values) are listed in Table S3. Finally, Figure S2 shows the volume changes of C_1_, C_2_, C_3_, C_4_ and H_2_ with respect to their respective values at the minimum energy geometry, as a direct function of the dihedral angle. At the planar barrier end, C_1_ and H_2_ clearly dominate the volume change. This is also true for the perpendicular barrier although here C_2_ beats H_2_ by a small margin. Overall, the changes are also less dramatic at this end of the total energy profile.

The next most powerful energy contribution that is working against the planar barrier is $V_{x}^{{{\rm H}_{2} {\rm H}_{2}^{'} }}$ (REG_i_ value = − 1.74), which is the fourth term to be discussed. Again, this term must necessarily represent a stabilising interaction along the barrier, when going from the equilibrium geometry to the planar one. This deduction makes sense because, as these two hydrogen atoms approach each other, they start engaging in a hydrogen–hydrogen bond [23]. There has been controversy in the literature about the nature of these interactions, that is, whether they are attractive or repulsive. From the point of view of IQA (and the REG analysis) there is no doubt: they are attractive. As the hydrogens approach each other, their (always negative) interatomic exchange energy becomes larger in magnitude. More precisely, as biphenyl becomes more planar, a weak covalent bond is forming between the hydrogens. Figure S3 shows how $V_{x}^{{{\text{H}}_{2} {\text{H}}_{2}^{'} }}$ changes with internuclear distance, between roughly 2 and 3.5 Å. This change is much pronounced than that of the companion atoms in the bay region, $V_{x}^{{{\text{C}}_{2} {\text{H}}_{2}^{'} }}$ and $V_{x}^{{{\text{C}}_{2} {\text{C}}_{2}^{'} }}$, also shown in Figure S3. The $V_{x}^{{{\text{H}}_{2} {\text{H}}_{2}^{'} }}$ values can be put in an even wider context. Figure S4 shows a logarithmic plot of *V*_X_^HH^ energies as a function of the internuclear distance for each of the 11 dihedral angle values. It is clear that the 1,6 interactions between the *ortho*-hydrogen H_2_ and H_2_′ are among the strongest of all possible H…H interactions. This type of plot was introduced before [30, 71] in order to cluster $V_{x}^{\rm AB}$ energies. Figures S5, S6 and S7, respectively, show, as a function of internuclear distance, all *V*_*X*_^CH^ and *V*_*X*_^CC^ energies for each of the 11 dihedrally controlled geometries and *V*_*X*_^AB^ for all interatomic interactions occurring in the energy minimum geometry only. Figure S6 shows that the terminal ends of the molecule (C_4_/H_4_) experience unusually strong (but still small absolutely speaking) exchange interactions in the planar geometry. This suggested a strong delocalisation of electron density across the molecule. Finally, Figure S7 puts $V_{x}^{{{\text{H}}_{2} {\text{H}}_{2}^{'} }}$ in the widest possible context and shows that it is a “non-covalent” (i.e. “through space”) interaction to be reckoned with.

Anticipating the overall discussion of Sect. 4 we can already see that the REG analysis is not confused by the coexisting and opposing behaviour of $E_{\text{intra}}^{{{\text{H}}_{ 2} }}$ and $V_{x}^{{{\rm H}_{2} {\rm H}_{2}^{'} }}$. Put more strongly, the REG analysis actually helps to resolve the apparent contradiction caused by this opposing behaviour. The $E_{\text{intra}}^{{{\text{H}}_{ 2} }}$ term expresses an energy destabilisation, that is, a steric clash between the ortho-hydrogens, while the $V_{x}^{{{\rm H}_{2} {\rm H}_{2}^{'} }}$ term expresses an energy stabilisation by forming a weak covalent bond between these hydrogens. The $E_{\text{intra}}^{{{\text{H}}_{ 2} }}$ term outweighs the $V_{x}^{{{\rm H}_{2} {\rm H}_{2}^{'} }}$ term. Both terms are well defined and again should not be confused in terms of explaining the overall planar rotation barrier. We come back to this point in Sect. 4, with the emergence of bond critical points between the two *ortho*-hydrogens. Note that any discussion has so far deliberately avoided bond critical points because their universal meaning is still controversial, in our opinion. However, IQA and the REG method *are independent of the presence or absence of bond critical points.*

We can go further and realise that this dual feature of stabilisation ($V_{x}^{{{\rm H}_{2} {\rm H}_{2}^{'} }}$) and destabilisation ($E_{\text{intra}}^{{{\text{H}}_{ 2} }}$) is actually a hallmark of bonds in homonuclear diatomics [12, 55]. Indeed, each hydrogen in H_2_ is destabilised by 33 kJ mol^−1^ while being part of H_2_ at its equilibrium geometry compared to the free atom. Of course, this destabilisation is massively compensated by the stabilising $V_{x}^{{{\rm HH}_{{}}^{'} }}$ energy contribution, which amounts to -630 kJ mol^−1^. In summary, an atom is destabilised when forming a homonuclear diatomic molecule, in the obvious absence of any charge transfer between the constituent atoms. Put differently, an atom “feels best” when it is on its own, free and isolated, compared to being inside the corresponding homonuclear diatomic. The perturbation of forming the diatomic increases the self-energy of each participating atom, which is disadvantageous from its own energetic point of view. As a final point, one may object that there is a little bit of charge transfer between an ortho-hydrogen and the carbon it is bonded to and thus conclude that $V_{\text{cl}}^{{{\rm H}_{2} {\rm H}^{\prime}_{2} }}$ should be included in the discussion. However, Figure S1 of the ESM takes away this potential objection. This figure shows the decomposition of $V_{\text{inter}}^{{{\rm H}_{2} {\rm H}^{\prime}_{2} }}$ into $V_{\text{x}}^{{{\rm H}_{2} {\rm H}^{\prime}_{2} }}$ and $V_{\text{cl}}^{{{\rm H}_{2} {\rm H}^{\prime}_{2} }}$. It is clear that $V_{\text{cl}}^{{{\rm H}_{2} {\rm H}^{\prime}_{2} }}$ is negligible compared to $V_{\text{x}}^{{{\rm H}_{2} {\rm H}^{\prime}_{2} }}$. This observation is also compatible with the minimal charge transfer in biphenyl (see above).

We now discuss the fifth and last interaction in the list of key energy terms, which is $V_{x}^{{{\rm C}_{1} {\rm C}_{2} }}$ having a REG_i_ of -4.59. Because of its negative value, this term works against the perpendicular barrier. By deduction, it is a stabilising energy, while approaching the perpendicular conformation. As a result, the four C_ipso_-C_ortho_ covalent bonds become stronger, and thus shorter, in that process. This deduction is confirmed by the bond length data showing a monotonic decrease from 1.396 (planar) to 1.390 Å (perpendicular).

Now that the list of five main energy contributions has been discussed, a few comments are due on the remaining energy terms. Starting with the planar barrier, $V_{x}^{{{\rm C}_{1} {\rm C}_{2} }}$ appears also here, as it did in the perpendicular barrier discussed just above. This mirroring behaviour, in which an energy term helps one barrier but opposes another barrier, occurs quite a number of times in Table 1. When a term shows this mirroring, it means that its chemical narrative extends over the whole energy profile. For example, in the case of $V_{x}^{{{\rm C}_{1} {\rm C}_{2} }}$, the energy profile of the C_ipso_-C_ortho_ (covalent) bonds fulfils two different roles depending on which side it is deployed: planar or perpendicular. In particular, $V_{x}^{{{\rm C}_{1} {\rm C}_{2} }}$ represents a continuously strengthening interaction in going from planar to perpendicular (i.e. more negative and hence stable at the perpendicular end). In summary, this single behaviour, throughout the whole energy profile, helps the planar barrier and opposes the perpendicular one. Further instances of mirroring energy contributions (visible in Table 1) occur for $E_{\text{intra}}^{{{\text{C}}_{ 1} }}$, $E_{\text{intra}}^{{{\text{C}}_{ 2} }}$, $V_{\text{x}}^{{{\rm H}_{2} {\rm H}^{\prime}_{2} }}$,$V_{x}^{{{\rm C}_{2} {\rm C}_{3} }}$,$V_{x}^{{{\rm C}_{2} {\rm H}_{2}^{'} }}$ or $E_{\text{intra}}^{{{\text{H}}_{ 2} }}$.

It becomes harder to discuss individual energy terms when they involve atoms further away from the central torsion angle because their effect becomes more subtle and may not connect readily with basic chemical knowledge. In particular, the interaction energies between atoms occurring in the two different phenyl rings are harder to understand chemically, such as $V_{\text{x}}^{{{\rm C}_{2} {\rm H}^{\prime}_{2} }}$. However, as the REG method is rooted in quantum mechanics, such terms play a non-negligible role from a physical point of view if their REG_i_ values are sufficiently high. However, such “cross-energy terms” are expected to be chemically more relevant in the case of ortho-substituted biphenyls, especially in the presence of large charge transfer (as caused by fluorine, for example). Finally, we point out that energy terms involving C_meta_ (i.e. C_3_) have smaller REG_i_ values than those involving C_ipso_ or C_ortho_, as expected.

Next we will move on to a second REG analysis, which is less resolved (i.e. coarser) than the REG analysis just discussed, now involving only single overall atomic energy (*E*_IQA_). However, we first present a useful extra analysis, which is not of REG nature and which is even coarser than the second REG analysis to follow. This extra analysis completely lacks atomic resolution, but operates at molecular level. Although useful, the analysis is actually a digression of the main narrative and hence appears in the ESM, as a full text with its own figures, namely Figures S8 and S9. In summary, we learn there that the *planar barrier is caused by the dominance of the intra*-*atomic destabilisation*, while the *perpendicular barrier results from the dominance of the interatomic destabilisation.* Hence, the *two energy barriers are each other’s opposite in character.*

### REG analysis: extracting chemical insight with a single overall atomic energy (E_IQA_)

The next question is which chemical insight is obtained when the resolution of the previous REG analysis is reduced to atomic resolution only. In other words, what do we learn if the three types of energy are added [see Eqs. (3) and (4)]? Table 2 shows the REG_*i*_ values of the 7 symmetry-unique atoms in biphenyl, for both its planar and perpendicular barrier. Table 2 is the equivalent of Table 1, but then for atomic resolution only (i.e. no information on energy type). It is instructive to analyse the barriers in this coarser way because one can argue that then the competition between the different energy types can be settled. In other words, the opposing behaviour of $E_{\text{intra}}^{{{\text{H}}_{ 2} }}$ and *V*_*x*_(H_2_–H_2_′), discussed at great length in the previous section, can then be reconciled by energy addition.

**Table 2:** The REG_i_ values of $E_{\text{IQA}}^{\rm A}$ (or E_IQA_(A)) for all seven symmetry-unique atoms in biphenyl

Planar barrier		Perpendicular barrier	
Energy term	REG	Energy term	REG
*E*_IQA_(C_2_)	1.13	*E*_IQA_(C_1_)	1.82
*E*_IQA_(C_3_)	0.46	*E*_IQA_(H_2_)	0.41
*E*_IQA_(C_4_)	0.21	*E*_IQA_(H_3_)	0.04
*E*_IQA_(H_2_)	0.19	*E*_IQA_(H_4_)	− 0.08
*E*_IQA_(H_4_)	0.03	*E*_IQA_(C_2_)	− 0.32
*E*_IQA_(H_3_)	− 0.07	*E*_IQA_(C_3_)	− 0.34
*E*_IQA_(C_1_)	− 0.91	*E*_IQA_(C_4_)	− 0.49

Note that $E_{\text{IQA}}^{\rm A}$ is the sum of the intra-atomic energy of atom *A* and the full interatomic potential energy involving all atoms, with which atom *A* interacts, other than *A.* Positive REG_*i*_ values assist the respective energy barrier, while negative REG_*i*_ values work against the barrier

**Fig. 5:**
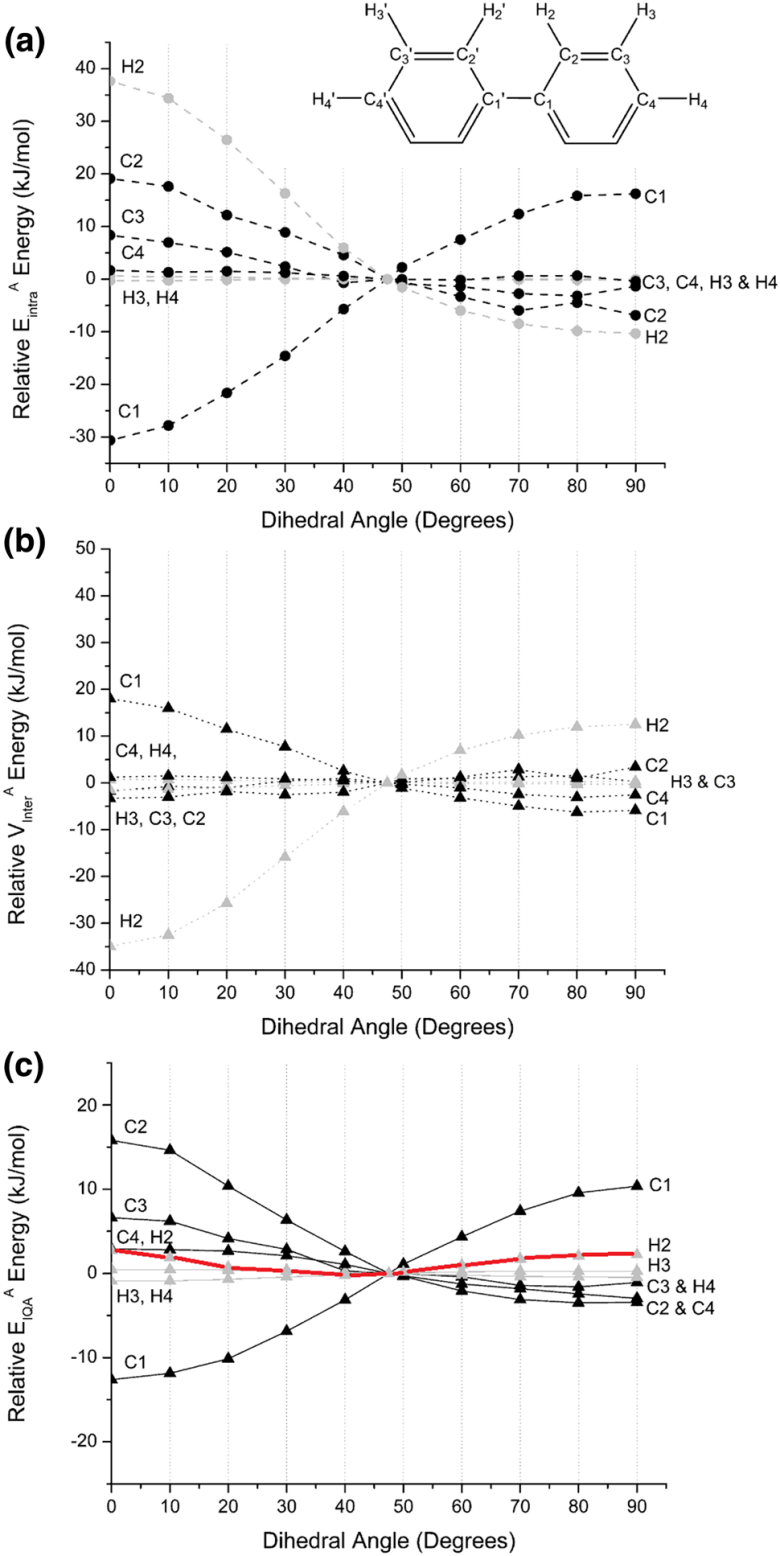
Variation at atomic resolution (atom *A*) in the (a) intra-atomic energies $E_{\text{intra}}^{\rm A}$, (b) interatomic energies $V_{\text{inter}}^{\rm A}$ and (c) the total atomic energies $E_{\text{IQA}}^{\rm A}$, relative to the values at the equilibrium geometry, of the 7 symmetrically unique atoms C_1_, C_2_, C_3_, C_4_, H_2_, H_3_ and H_4_ (see upper half of the right phenyl ring) plotted against the torsion angle. Carbon and hydrogen profiles are drawn in black and light grey, respectively. Each energy profile is multiplied by the number of occurrences (of a symmetrically unique atom) according to the molecular symmetry. In view of the literature’s special attention to the ortho-hydrogens, a red curve marks the energy profile of the atoms symmetrically related to H_2_ (i.e. all four ortho-atoms H_2_′, H_6_ and H_6_′)

Starting with the planar barrier it is now clear that the *ortho*-hydrogen H_2_ loses the prominent role it had in the energy-type resolved REG analysis. The REG_*i*_ value is ranked merely fourth and amounts to only 0.19. In order to explain this result one suspects, of course, that $E_{\text{intra}}^{{{\text{H}}_{ 2} }}$ and *V*_*x*_(H_2_–H_2_′) largely cancel each other out. The evidence for this suspicion is provided in Fig. 5, which shows the atomic fine structure via three panels: (a) intra-atomic energies $E_{\text{intra}}^{\rm A}$, (b) interatomic energies $V_{\text{inter}}^{\rm A}$ and (c) the total atomic energies $E_{\text{IQA}}^{\rm A}$. Again we need to show only 7 out of 22 atoms in total. Because of molecular symmetry, only about a third of the atoms need to be monitored.

A first important observation following Fig. 5a concerns the large differences in magnitude between the various symmetrically unique atoms: the profiles of C_4_, H_3_ and H_4_ are negligible, while those of the bay (ortho-) atom H_2_ and the central atom C_1_ are dominant, each in their own way. Secondly, all non-negligible energy profiles have qualitatively the same shape as the overall summed intra-atomic energy of Figure S8, except that of C_1_. Indeed, the contribution of C_1_ to the overall intra-atomic energy *stabilises* the system (by 15.3 kJ mol^−1^ at 0°, equalling half the value read off for the C_1_ curve in Fig. 5a) at the lower torsion angles, but reduces stability (by 8.1 kJ mol^−1^ at 90°, again half the value) at torsion angles larger than that of the energy minimum. Thirdly, the profiles decrease in magnitude for atoms further away from the central torsion pivot (C_1_). Indeed, the three carbon profiles (C_2_, C_3_ and C_4_) monotonically decrease in amplitude as the carbons move away from C_1_. This behaviour is somewhat expected because the further a carbon is away from the centre of the geometrical perturbation, the less it would be affected. This chemically intuitive order is also respected in the ranking of E_IQA_(A) in Table 2: C_2_ (C_ortho_), C_3_ (C_meta_) and C_4_ (C_para_).

Next we discuss Fig. 5b, which offers atomic resolution of the coarse-grained interatomic energy $\sum\nolimits_{A} {\sum\nolimits_{B \ne A} {V_{\text{inter}}^{\rm AB} } }$ shown in Figure S8. The shorthand notation $V_{\text{inter}}^{\rm A} = \frac{1}{2}\sum\nolimits_{B \ne A} {V_{\text{inter}}^{\rm AB} }$ (used in Eq. 4) is convenient to denote the energy that a given atom *A* experiences while interacting with all other atoms in biphenyl except itself. First, it is clear that H_2_ and C_1_ are again dominant, and even more so when contrasted with their previous dominance (relative to other atoms) in Fig. 5a. Indeed, in Fig. 5b the profiles of not only C_4_, H_3_ and H_4_ are negligible, but also those of C_2_ and C_3_. Again, as before in Fig. 5a, the profiles of C_1_ and H_2_ mirror each other and H_2_ dominates C_1_ once more. Hence, H_2_ determines the behaviour of the overall interatomic energy profile in Figure S8. Secondly, the amplitude of the energy profile is larger for small torsion angles, near the planar conformation. This observation is possibly explained by the general 1/R dependence of this type of energy, where *R* is an internuclear distance. Indeed, the internuclear distances between a given atom *A* and all its neighbours are smaller, and hence 1/R larger, for small torsion angles.

Next, we discuss Fig. 5c where the effects of Fig. 5a, b are brought together, through summation (see Eq. 4) leading to *E*_IQA_(A). The red energy profile highlights the extent of the cancellation between the intra-atomic and interatomic energies for the H_2_ atoms. The C_2_ and C_3_ atoms constitute the most dominant part of the planar barrier, but are offset by the opposing contribution of C_1_. The perpendicular barrier is largely caused by the C_1_ contribution alone. This observation is compatible with the fact that *E*_IQA_(C_1_) has the most negative REG_i_ value in Table 2, compatible with its nature of working most against the planar barrier. Similarly, *E*_IQA_(C_1_) helps most in constructing the perpendicular barrier, as demonstrated in Fig. 5c and the top entry in Table 2.

### REG analysis: background IQA terms

Sections 3.1 and 3.2 both focus on the first aim of the REG method, namely extracting chemical insight from an energetically partitioned system. The current section focuses on the second aim of determining *subsets* of partitioned energies that best describe how the total energy changes over the total energy profile. An IQA analysis partitions the total energy of a system into energy contributions, and the total energy profile is recoverable from the summation of these energy contributions. Section 2.4 explains that there are 484 IQA terms for biphenyl, when allowing for the usual three types of energy contribution. With this in mind, we can group energy contributions into two subsets. The first subset consists of the “*foreground*” energies, which most determine the behaviour of the total system according to the REG method. The second subset consists of the “*background”* energies, none of which contribute significantly to the total behaviour of the system. Individually, the energy contributions in the foreground subset will have large-magnitude REG_i_ values, while energy contributions in the background subset will have small-magnitude REG_i_ values. In order to decide which of the energy contributions should be included in *both* the foreground and background subsets, we must determine a cut-off magnitude for the REG_i_ values. Energy contributions with REG_i_ value magnitudes larger than this cut-off will be in the foreground subset. Conversely, energy contributions with REG_i_ value magnitudes smaller than this cut-off will be placed into the background subset. As such, one strives for chemical insight to be obtained from the foreground subset only.

**Fig. 6:**
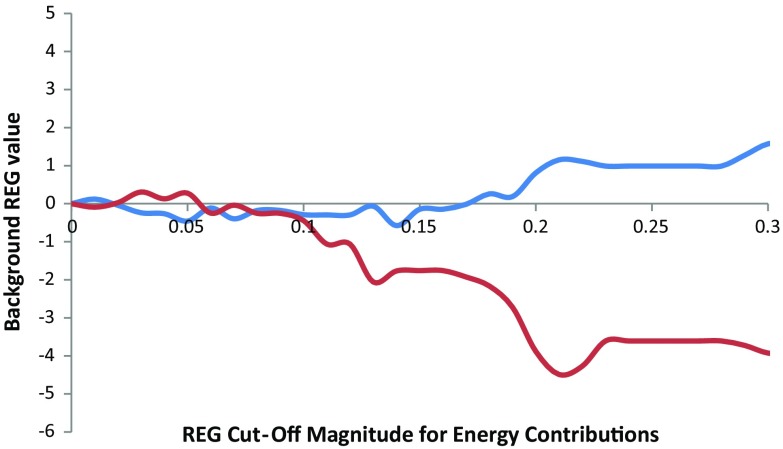
The background REG value against the REG cut-off value. The blue line represents the background REG value of the planar barrier, and the red line represents the background REG value of the perpendicular barrier

Figure 6 is introduced in order to determine the REG cut-off magnitude that is required to define the two subsets. Figure 6 shows how the background REG value for each barrier changes as the cut-off magnitude is increased. The background REG value is calculated by the application of Eq. (6) to the sum of all energy contributions with REG magnitudes less than the cut-off. As such, a cut-off magnitude of zero coincides with no energy contributions being included in the background. It is desirable to include as many energy contributions in the background as possible, while enforcing the constraint that the background should not contribute significantly to the behaviour of the total system. Put explicitly, a cut-off REG magnitude should be maximised while keeping the background REG value close to zero. By considering Fig. 6, we can see that the background REG of each barrier starts to diverge from zero at a cut-off (absolute) value of about 0.1. This shows that REG cut-off magnitudes greater than 0.1 begin to include “chemically meaningful” energies in the background, which is undesirable. Therefore, the REG cut-off magnitude of 0.1 is used here to distribute the energy contributions over the foreground and background subsets. In other words, the definition of a background energy contribution is that it has an absolute REG_i_ value of less than 0.1 for both the planar and the perpendicular barriers. Put differently yet again, a background energy contribution has an absolute REG_i_ value of less than 10% that of the total REG_i_ value in all the segments of the PES. Hence, any energy contribution that is not considered a background term shall, by this definition, be deemed a foreground term.

**Fig. 7:**
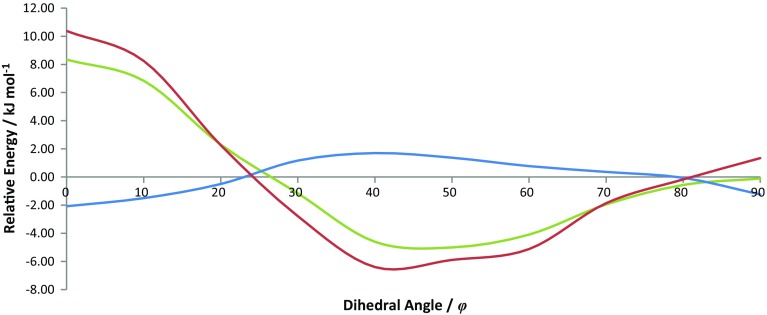
The PES formed by the background IQA terms, foreground IQA terms and the total energy PES: total PES (green), background PES (blue) and foreground PES (red)

Figure 7 shows the foreground and background PES when the REG cut-off has been applied to the energy partitioned terms and compares it to the total energy PES. It should be noted that a partitioned energy is assigned to the foreground subset if it has a REG magnitude greater than the cut-off in any barrier.

Figure 7 shows that the foreground PES matches quite closely the total energy. This observation indicates that the foreground subset contains the majority of energy contributions required to recreate the total behaviour of the system. On the other hand, the background PES (blue) shows little to no behaviour as the PES is reasonably flat. However, the background PES acts to dampen the foreground PES because the former shows inverse behaviour to the foreground PES. This dampening effect can be due to the accumulation of small energy changes that are individually insignificant, but overall lead to a behaviour that opposes that of the system. Although all the background energies are independently small the total sum of these energies may not be small. Therefore, the sum of all the background energies may provide an overall effect on the PES (for example dampening), which is qualitatively important.

The definition given for a background energy contribution is such that this subset is the same for each barrier (i.e. the energy contributions in the background subset are always the same regardless of the barrier). For biphenyl, there are 414 background terms, meaning that 86% of all energy contributions are background terms. The REG values due to these background terms are $m_{\text{REG,background}}^{{{\text{planar}}\;{\text{barrier}}}} = -\,0.29$ and $m_{\text{REG,background}}^{{{\text{perp}} .\;{\text{barrier}}}} = -\,0.44$. Individually, background terms are small (less than 10% of the total force), but the total REG value provided by the background terms is significant, with magnitudes that are 29% and 44% of the total REG value for the planar and perpendicular barriers, respectively. This observation provides evidence that the background REG value in chemical systems may have significant damping or amplifying effects. Hence, in order to quantitatively recreate the behaviour of a system, every atomic and energetic contribution must be considered. Each of the barrier’s REG values is negative, showing that the background is relatively unstable around 50°, which can be verified by inspecting Fig. 7. This figure shows that, qualitatively, the behaviour of biphenyl can be described by only 70 (14%) of the total number of energy contributions because the foreground PES and total PES show the same trend in energy.

## Overall discussion

The original controversy over the origin of the planar barrier in biphenyl actually started with the appearance of a bond critical point between the *ortho*-hydrogens, in geometries with a central torsion value of less than 28°. Figure S10 shows the appearance of these bond critical points in the planar geometry. The actual meaning of a bond critical point is still an open problem, which will take much time to solve satisfactorily because it has been discussed from so many angles; a final solution will have to address all case studies and make sure that all falls in place. Strictly speaking, the current study does not impinge on the controversy surrounding the meaning of bond critical points. Indeed, both the REG and IQA methods operate on continuously varying energy contributions and their conclusions do not depend on the discontinuous presence or absence of bond critical points. However, in the case of biphenyl, the REG method can shed light on the psychology of the confusion between H…H bonding and steric hindrance, both involving the *ortho*-hydrogens.

Let us focus on the planar barrier and recall what the REG analysis is telling us here. REG is able to handle competing effects, which it ranks by a simple algorithm. The analysis is automatic and involves literally all energy contributions, even if they run into hundreds. The ranking is based on the computer program ANANKE inspecting all possible ratio of slopes, that is, a slope of a given energy contribution and that of the total energy. These slopes exist because the REG analysis is fundamentally *dynamic*: it cannot judge a snapshot of a system; instead, it needs a change in the various energies, as controlled by the torsion angle (in this case). The central idea of REG is that the energy contribution that changes most dramatically along the same lines as the total energy offers the chemical insight we are looking for. It is this energy contribution and the chemical insight associated with it that governs the total energy profile (over a particular segment). Of course, there will also be an energy contribution that acts most dramatically against the total system’s energy behaviour. However, REG can cope with competing and conflicting contributions without getting confused.

Introducing the metaphor of analysing a football match is helpful at this point. An energy contribution equates to a player and the total system’s energy equates all players on the field. The actions of all players are “added up” to yield an overall football game, which is similar to the addition of all energy contributions in chemistry, leading to an overall energy profile. Now, the scoring of a goal is a particular phenomenon occurring in the game. This goal corresponds to a rotation barrier, a (chemical) phenomenon occurring in the total energy profile. The question is now how one can *explain* this goal. Even in the complex situation of a typical game one can still meaningfully say that a particular player scored a goal. Does one look at each player’s contribution, no matter how minute, and distribute the “explanation” over all their actions? Does one just look at each of the two opposing teams as a whole and give a broad explanation of why one team managed to score that goal? Or does one give up on finding the explanation and simply say that it emerged somehow from both teams interacting? Or does one focus on the last action of one or two players just before the goal, fast and dramatic as it was? The answer is typically “yes” to the last option. Indeed, this type of analysis is carried out by thousands of commentators and millions of spectators. Equally, the REG method can reveal the player who made that goal, where the fast, last-second action corresponds to the highest-ranking REG value. The REG method can reveal the player even if other players tried to prevent the goal. This is the REG analysis at atomistic level; the method does not get confused by how a goal emerges from the interaction of two opposing players.

The football metaphor can be pushed further, but let us now return to chemistry, in particular, to biphenyl. We remind ourselves that two REG analyses were carried out: (1) resolved both by atom and by energy type, and (2) resolved by atom only. The former analysis told us that the planar barrier is most supported by the rise in intra-atomic energy of the *ortho*-hydrogens towards planarity, i.e. a steric clash. This is indeed what textbooks state. *Some papers made the error of associating this repulsive interaction with a bond critical point.* Instead, the bond critical point should be associated with the attractive exchange interaction between the two *ortho*-hydrogens. IQA crisply separates the two types of energy contribution, and the REG method acts on this sharp distinction. The attractive interaction becomes stronger all the way from the perpendicular geometry towards the planar geometry. This stabilising interaction coexists with the steric interaction, which also grows monotonically from the perpendicular geometry towards the planar geometry. Our view does not agree with that of Poater, Solà and Bickelhaupt who published a paper [24] with the title “*Hydrogen*–*hydrogen in planar biphenyl* (…) *does not exist*”. Our analysis agrees with that [29] of Eskandari and van Alsenoy, and that [72] of Hernández-Trujillo and Matta, who independently confirmed hydrogen–hydrogen bonding in planar biphenyl. In summary, the bond critical point controversy that set the study of biphenyl in motion is resolved for this case study: it is the product of confusing two opposing and coexisting interactions.

However, one can now argue that if two strong but opposing energy contributions are added, then they largely cancel. In that case, perhaps another un-cancelled energy contribution will offer the chemical explanation for the barrier. Metaphorically, if two football players (forward and defender) cancel their actions in front of the goalkeeper, then perhaps a third player scores the goal. Actually, this is what happens in the case of the planar biphenyl barrier. The second type of REG analysis, resolved by atom only, finds that C_ortho_ (via *E*_IQA_(C_2_)) is the main cause for the barrier. This conclusion is confirmed [29] by Eskandari and van Alsenoy. However, they point out that the virial-based atomic energy (which predates IQA) of C_ipso_ (i.e. C_1_) supports the planar barrier, which contradicts the result given by IQA. Indeed, our REG analysis also states that the intra-atomic energy of C_ipso_ works *against* the planar barrier rather than in favour of it. Hence, we cannot agree with the previously published [72] conclusion that the “lengthening of the C_ipso_–C_ipso_ bond (…) in planar biphenyl results in a destabilisation of these atoms”. To the contrary, we find that C_ipso_ is *stabilised* in the planar geometry. Finally, we note that Dillen confirms [73] that the traditional virial-based atomic energy is lower for atoms in congested molecules, whether they are hydrogen or fluorine atoms. This observation goes against the intuition that if atoms are brought too close together they must pay an energetic penalty. IQA does return this penalty and hence contrasts with the virial-based energy. In summary, virial-based energies at stationary points are nothing but minus kinetic energies, and the latter rise steeply (in absolute value) in sterically crowded environments where atomic volumes decrease. Hence, virial energies in steric clashes would almost always favour the congested environments, which cannot be right.

The coarsest analysis of the planar barrier is non-REG and non-atomistic. It simply says that the planar barrier is caused by the dominance of overall intra-atomic energy. In other words, at the planar geometry the atoms that feel worse off individually dominate.

One can ask which level of interpretation is correct: (1) non-REG and non-atom, (2) energy-type REG or (3) atom-only REG. The football metaphor comes in handy again. It is possible to analyse a match without referring to individual players. However, a coach should always keep in mind what his players are worth and how they may act out during a match. The action of a given player may well be cancelled by that of an opposing player. Replace this player and a particular goal may well take place. Similarly, a chemist (i.e. the coach) should keep in mind what the individual atoms are doing in his or her molecule. It is profitable to be aware that the two *ortho*-hydrogens are *actually reducing* the planar barrier through their exchange interaction. Substituting the *ortho*-hydrogen for a chlorine (i.e. substituting a player) should then not be naively seen in terms of steric interaction only. The knowledge that substituents may exchange a lot may tip an energy barrier to an unexpected and counterintuitive side.

The closing discussion on the perpendicular barrier is briefer because (1) it never suffered from the confusion caused by a wrong interpretation of a bond critical point and (2) the lack of literature on this barrier means that there are no comparing notes. In a nutshell, the two C_ipso_ atoms dominate this barrier, by the lack of stabilising exchange energy between them in the perpendicular geometry. However, their intra-atomic energy is also very unfavourable at the barrier.

## Conclusion

The minimal and reference-independent energy partitioning scheme IQA has the capacity to separate stereo-electronic effects into three well-defined energy contributions: intra-atomic (steric), exchange (covalent) and electrostatic. The REG method is an exhaustive procedure that automatically ranks atomic energy contributions according to their importance in explaining the energy profile of a total system. The REG-IQA method analyses for the first time *both* the planar and perpendicular energy barriers in biphenyl.

The planar barrier is caused by the inner destabilisation of the two *ortho*-hydrogens, which is equivalent to the textbook steric clash. However, this destabilisation is partially counteracted by the formation of a very weak covalent bond between the *ortho*-hydrogens. When energy types are summed, this partial cancellation diminishes the role of the *ortho*-hydrogens. The REG analysis then identifies, at this overall atomistic level, that C_ortho_’s energy behaviour is the cause of the planar barrier.

The perpendicular barrier is explained by the energy behaviour of the C_ipso_ atoms. When resolved by energy type, the lack of stabilising exchange energy between the two C_ipso_ atoms predominately causes this barrier, as well as their reduced internal energy.

## Electronic supplementary material

Below is the link to the electronic supplementary material.

Supplementary material 1 (DOCX 925 kb)
