# Supplementary material for: A relative energy gradient (REG) study of the planar and perpendicular torsional energy barriers in biphenyl
Source: Theor Chem Acc. 2018 Dec 10;138(1):12. doi: 10.1007/s00214-018-2383-0 (PMC6383956; doi:10.1007/s00214-018-2383-0)
Supplement: Supplementary file 1 — Supplementary material 1 (DOCX 925 kb) [file 214_2018_2383_MOESM1_ESM.docx]

**Electronic Supplementary Material (ESM)**

**A Relative Energy Gradient (REG) study of the planar and perpendicular torsional energy barriers in biphenyl**

Paul LA Popelier*, Peter I Maxwell, Joseph CR Thacker and Ibon Alkorta^+^

Manchester Institute of Biotechnology (MIB), 131 Princess Street, Manchester M1 7DN, Great Britain and School of Chemistry, University of Manchester, Oxford Road, Manchester M13 9PL, Great Britain

^*^Corresponding Author: [pla@manchester.ac.uk](mailto:pla@manchester.ac.uk), +44 161 3064511

^†^Instituto de Química Médica (IQM-CSIC), Juan de la Cierva, 3, 28006 Madrid, Spain

**Tables**

| Dihedral Angle  (degrees) | ΔE  (kJ/mol) |
| --- | --- |
| 0 | -0.27 |
| 10 | -0.36 |
| 20 | 0.23 |
| 30 | -0.48 |
| 40 | 0.32 |
| 47.6 | 0.38 |
| 50 | 0.09 |
| 60 | 0.29 |
| 70 | -0.10 |
| 80 | -0.05 |
| 90 | 0.03 |

**Table S1.** Energy discrepancy (ΔE) between the total molecular energy and the energy obtained by summing all intra- and interatomic IQA energy contributions. The absolute value of this error never exceeds 0.5 kJ/mol, which is 4 % or 9 % of the planar or perpendicular torsional energy barrier, respectively. All values are also plotted in Figure 2 of the main text, as a dashed green line.

|  | Type | Range | | | |
| --- | --- | --- | --- | --- | --- |
| Interaction |  | **Distance** | **V_inter_^AB^** | **V_x_^AB^** | **V_cl_^AB^** |
| C1-C1' | 1,2 | 0.0093 | 5.8 | 9.7 | 2.7 |
| C1-C2 | 1,2 | 0.0054 | 5.1 | -5.4 | 5.7 |
| C2-C3 | 1,2 | 0.0020 | 2.3 | 2.7 | 5.4 |
| C3-C4 | 1,2 | 0.0016 | 0.4 | 0.4 | 5.3 |
| C2-H2 | 1,2 | 0.0039 | 1.3 | 0.1 | 1.2 |
| C3-H3 | 1,2 | 0.0001 | 0.1 | 0.04 | 0.2 |
| C4-H4 | 1,2 | 0.0003 | 1.5 | 0.7 | 0.1 |
| C1-C2' | 1,3 | 0.0212 | 1.8 | -0.8 | 0.5 |
| C1-C3 | 1,3 | 0.0233 | 0.7 | 0.5 | 0.2 |

**
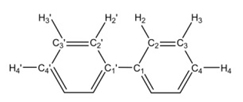
**

**Table S2.** Distance and interatomic energy ranges of all seven unique (by molecular symmetry) 1,2 interactions, and alongside two 1,3 interactions. Distances are given in Å and energies in kJ/mol.

|  |  | **C_1_** |  |  |  | **H_2_** |  |
| --- | --- | --- | --- | --- | --- | --- | --- |
| **Dihedral Angle** | **0.0004 au** | **0.001 au** | **0.002 au** |  | **0.0004 au** | **0.001 au** | **0.002 au** |
| **0** | 82.87 | 72.92 | 64.67 |  | 58.80 | 45.66 | 36.07 |
| **10** | 82.58 | 72.78 | 64.56 |  | 59.11 | 45.85 | 36.19 |
| **20** | 81.83 | 72.24 | 64.28 |  | 59.96 | 46.38 | 36.48 |
| **30** | 80.71 | 71.60 | 63.91 |  | 61.23 | 47.13 | 36.92 |
| **40** | 79.49 | 70.93 | 63.58 |  | 62.72 | 47.98 | 37.38 |
| **47** | 78.44 | 70.39 | 63.38 |  | 63.91 | 48.56 | 37.57 |
| **50** | 78.13 | 70.26 | 63.32 |  | 64.21 | 48.70 | 37.61 |
| **60** | 77.12 | 69.95 | 63.32 |  | 65.32 | 49.23 | 37.65 |
| **70** | 76.23 | 69.67 | 63.45 |  | 66.04 | 49.37 | 37.56 |
| **80** | 75.38 | 69.53 | 63.66 |  | 66.33 | 49.32 | 37.43 |
| **90** | 75.14 | 69.42 | 63.73 |  | 66.38 | 49.35 | 37.38 |

**Table S3.** Dependence of the volumes of atom types C_1_ and H_2_ on the central dihedral angle. Volumes for three possible outer boundaries of the topological atoms are given, corresponding to constant electron density envelopes of the whole molecule, set at contour values of 0.0004, 0.001 and 0.002 a.u.

Table S3 shows that the volume of H_2_ increases from its minimum value at the planar geometry towards the perpendicular geometry. This atom keeps expanding while its intra-atomic energy decreases until, at the end of its trajectory (90^o^), it is 7 % larger than at the start (0^o^). In other words, atom H_2_ starts in a congested environment that destabilises it. As the torsion angle increases the atoms around H_2_ recede, the congestion eases and the atom H_2_ stabilises until it reaches its lowest intra-atomic energy at 90^o^.

The C_1_ atom follows the same principle but in the opposite direction: at 0^o^ it has the largest volume and shrinks as the biphenyl moves towards its perpendicular conformation. The shrinking of the C_1_ atoms again corresponds to a congestion process in its immediate environment. This congestion is dominated by the fluctuation of the C_1_-C_2_ interaction in which the bond shortens with increasing torsional angle, allowing this atom to stabilise itself. The alternative competing bond is the C_1_-C_1_’, which has its shortest distance in the equilibrium geometry and elongates with torsional increase or decrease.

As a final technical remark, the volume analysis is independent of the 0.001 a.u. value since the alternative values of 0.002 a.u. and 0.0004 a.u. lead to the same interpretation. The observations made here are directly in line with those identified by Dillen [[1](#_ENREF_1)] whereby internuclear distance, atomic volume and $E_{\text{intra}}^{A}$ are all interlinked.

**Figures**


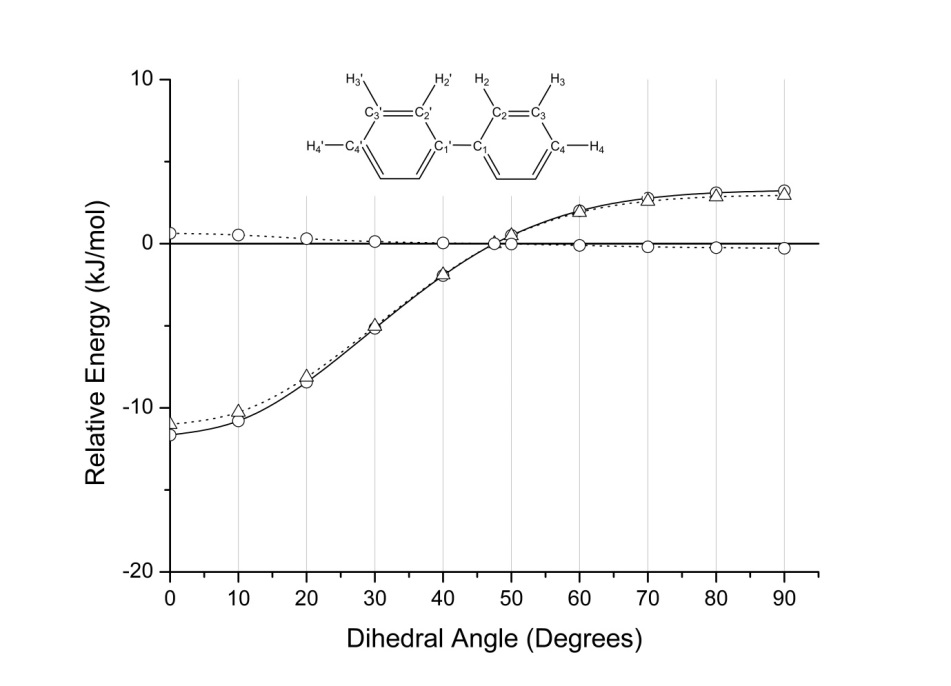


**Figure S1.** Decomposition of $V_{\text{inter}}^{H_{2}H'_{2}}$(triangles) into $V_{\text{x}}^{H_{2}H'_{2}}$(solid circles) and $V_{\text{cl}}^{H_{2}H'_{2}}$(dotted circles), all plotted relative to the equilibrium geometry. Within biphenyl, the supremacy of the role of $V_{\text{X}}$over that of $V_{\text{cl}}$is also highlighted from other key energy contributions.


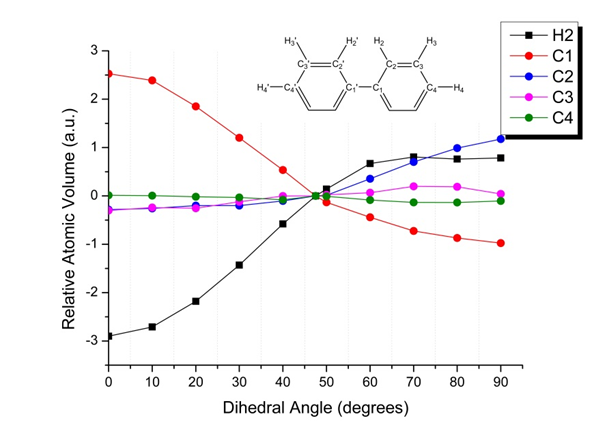


**Figure S2.** Relation between the atomic volume (for 0.001 a.u. constant electron density envelope) and dihedral angle in the molecule. All volumes are expressed with respect to their respective value at the equilibrium geometry.


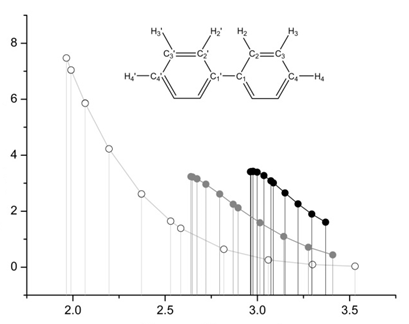


**Figure S3.** The exchange energy V_X_^AB^ (in kJ/mol) between atoms in the bay region (H_2_…H_2_’, C_2_…H_2_’ and C_2_…C_2_’) and a function of internuclear (AB) distance (in Å). Carbon-carbon interactions are black, carbon-hydrogen interactions grey and hydrogen-hydrogen interactions white. Each energy profile features one example of an interaction, so symmetry multiplicity is not accounted for.


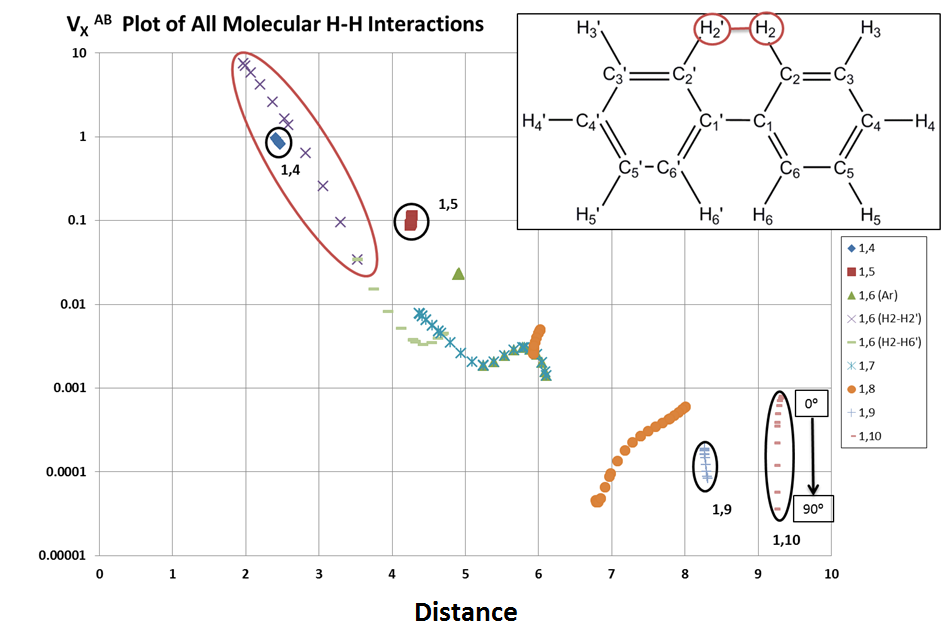


**Figure S4.** Logarithmic plot of V_X_^HH^ energies (kJ/mol) *versus* internuclear H…H distance (in Å) for each dihedral angle (11 values for each energy, marked by a symbol in the legend). It is clear that the 1,6 interactions, between the *ortho* hydrogen H_2_ and H_2_’ are amongst the strongest of all. The overall broad linearity of the logarithms of the energy with respect to internuclear distance is typical for this plot and the two following plots. We introduce the notation 1,*n* where 1 and *n* represent atoms that are separated by *n*-1 covalent bonds.

**
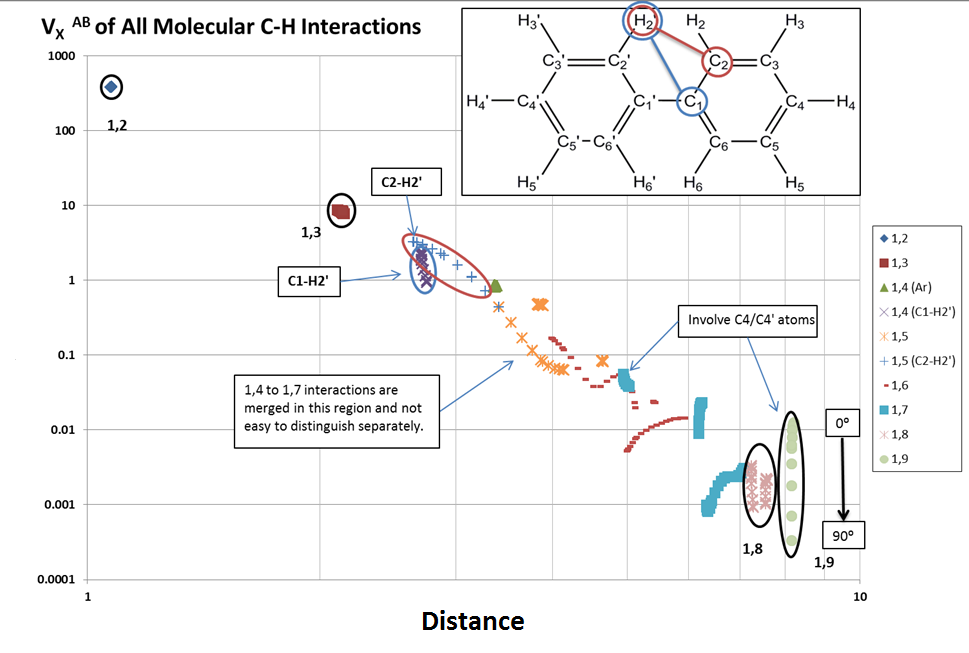
**

**Figure S5.** Logarithmic plot of V_X_^CH^ energies (kJ/mol) *versus* internuclear C…H distance (in Å) for each dihedral angle (11 values for each energy, marked by a symbol in the legend). The structural covalent backbone is seen in the 1,2 interactions in the upper-left most quadrant in the figure. The scale prevents any obvious fluctuations to be easily identified.


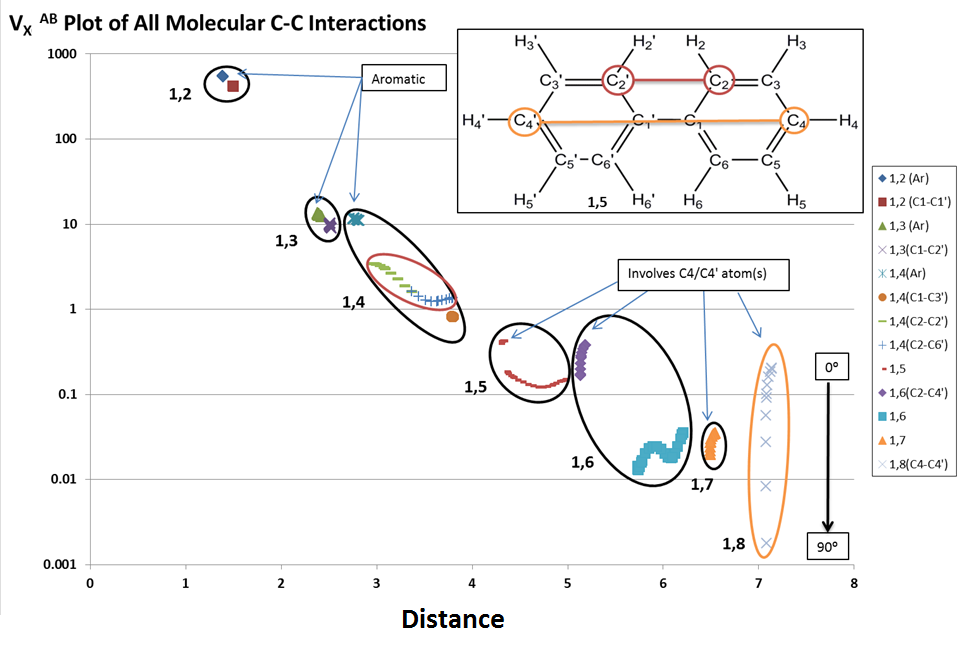


**Figure S6.** Logarithmic plot of V_X_^CC^ energies (kJ/mol) *versus* internuclear C…C distance (in Å) for each dihedral angle (11 values for each energy, marked by a symbol in the legend). The C_2_-C_2_’ (encircled red) and C_4_-C_4_’ (encircled orange) interactions are highlighted as indicated on the inset molecule.

Most interestingly is the behaviour of the 1,8 C_4_-C_4_’ interactions (encircled orange and also highlighted in inset diagram). In the planar geometry (0^o^ C), a much stronger exchange interaction is formed between the atoms, giving it the same magnitude of stability as commonly seen in the 1,5 interaction category. This stability is lost during torsional rotation towards the perpendicular geometry, where it reaches the stability expected for a 1,8 interaction, and in-keeping with the typical overall linear behaviour of V_X_^AB^ *versus* distance. This distinctive behaviour is also present for the 1,9 C_4_-H_4_’ and 1,10 H_4_-H_4_’ interactions in Figure S4 and S5.

**
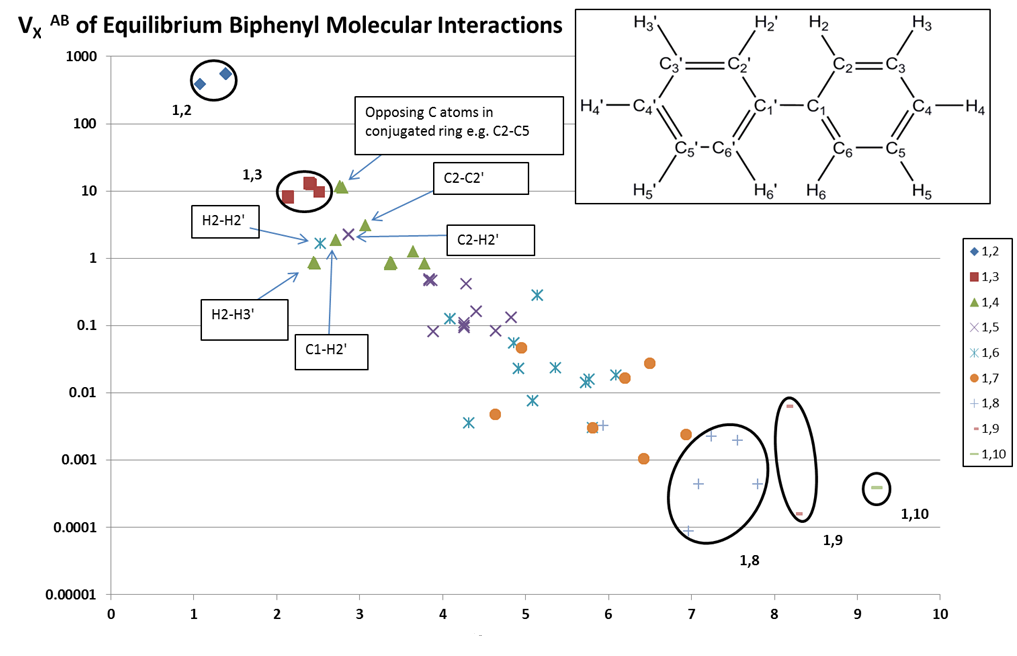
**

**Figure S7.** Logarithmic plot of all V_X_^AB^ energies (kJ/mol) *versus* all internuclear distances (in Å) but only for the energy minimum geometry.

**A coarse-grained non-REG analysis, at molecular level**


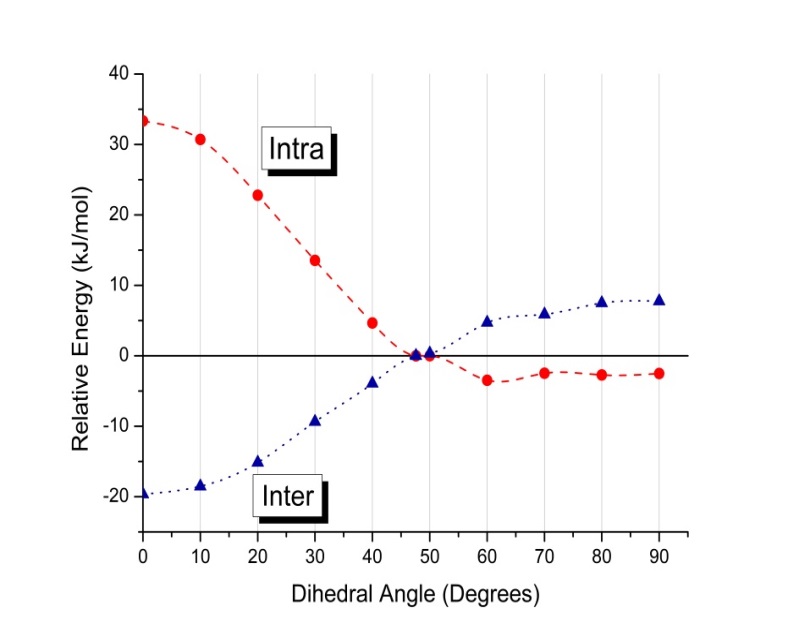
 A coarse-grained QCT partitioning offers preliminary insight in the very different nature of the planar energy barrier compared to the perpendicular one. Figure S8 shows the *overall* intra-atomic energy summed over all atoms (including all symmetrically equivalent atoms),$\sum_{A} E_{\text{intra}}^{A}$, and the *overall* inter-atomic energy summed over all possible atom-atom pairs, $\sum_{A} \sum_{B\neq A} V_{\text{inter}}^{AB}$.

**Figure S8.** A coarse-grained breakdown of energy into overall intra- and interatomic contributions. Profiles of the *overall* intra-atomic energy (summed over all atoms) (red circles) and the overall inter-atomic energy (summed over all atoms) (blue triangles). All energies are relative to the equilibrium geometry. The zero dihedral angle τ corresponds to the planar conformation. The sum of the intra-atomic and interatomic energy at τ = 0^o^ results in the planar energy barrier of 13.7 kJ/mol (see Figure 2).

Figure S8 shows how the overall intra-atomic energy (red circles) decreases monotonically from the planar conformation towards the equilibrium geometry and then stays more or less stationary beyond this point. This means that, overall, certain atoms start from a congested situation at the planar conformation and gradually relax (i.e. stabilise) towards the equilibrium geometry, after which their energetic stabilisation stagnates.

The overall inter-atomic energy almost mirrors the intra-atomic energy. The inter-atomic energy *increases* monotonically from its lowest point at the planar conformation, and again (almost) stagnates beyond the equilibrium geometry. The two profiles annihilate each other, were it not for a relatively small but substantial discrepancy: the *planar barrier* *is caused by the* *dominance of the intra-atomic destabilisation* while the *perpendicular barrier results from the dominance of the inter-atomic destabilisation.* Hence the *two energy barriers are each other’s opposite in character.*

We now refine the previous coarse-grained analysis by first breaking down the *overall* intra-atomic energy ($\sum_{A} E_{\text{intra}}^{A}$) into its three components, each being a sum over all atoms: kinetic energy, electron-electron repulsion energy and electron-nucleus attraction energy (see Eqn. 1 of the main text for one atom, but here summed over *A*:$\sum_{A} T_{A},\sum_{A} V_{ee}^{AA},\sum_{A} V_{en}^{AA}$).

Figure S9 shows the result. Compared to its value at the equilibrium geometry, the kinetic energy barely varies with the central torsion angle, by a few kJ/mol only. However, the electron-electron repulsion and electron-nucleus attraction energies vary much more, and almost cancel each other. Both types of energy are up to two orders of magnitude larger than that of the kinetic energy.


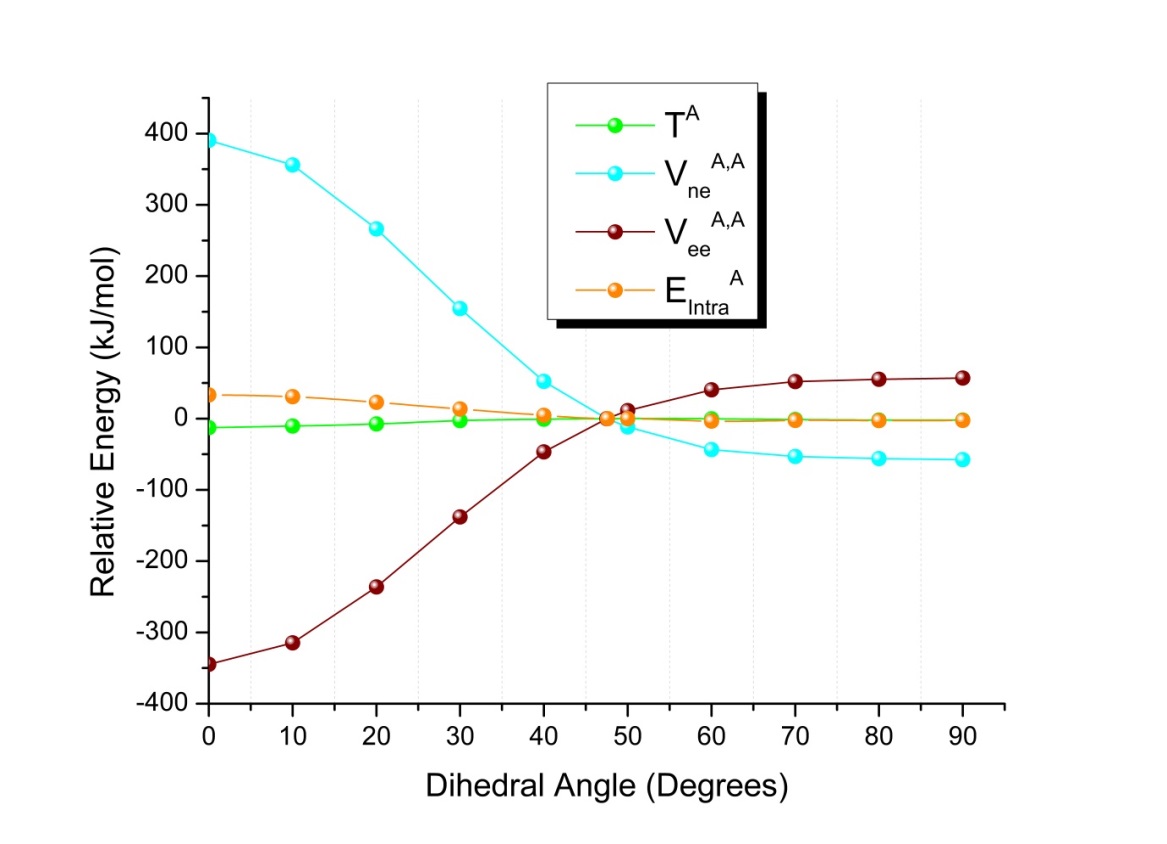


**Figure S9.** Breakdown of the overall intra-atomic energy, $\sum_{A} E_{\text{intra}}^{A}=\sum_{A} (T_{A}+V_{ee}^{AA}+V_{en}^{AA})$ into its three components (summed over all atoms): kinetic energy, electron-electron repulsion energy and electron-nucleus attraction energy. All energies are relative to those at the equilibrium geometry. Note that the $\sum_{A} E_{\text{intra}}^{A}$(orange) profile is the same as the red profile in Figure S8.

In summary, at the planar energy barrier the overall electron-nucleus attraction energy destabilises the molecule more than the electron-electron repulsion does, compared to the equilibrium geometry. At the perpendicular barrier the opposite is true. This conclusion is chemically not intuitive because a typical chemist does not think in terms of the three energy components discussed above. This is why it is best to bundle the three components into a single intra-atomic energy and aim to make sense of this energy’s behaviour in a chemically intuitive way. We will see (in the main text) that steric congestion provides an intuitive interpretation but this becomes only clear at the level of individual atoms.

| 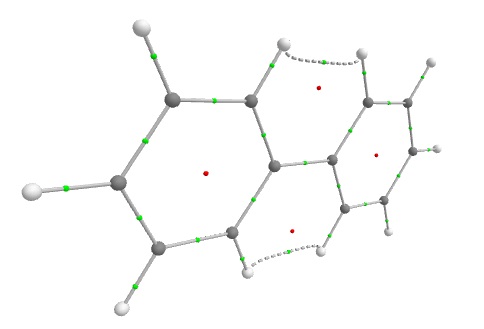 | 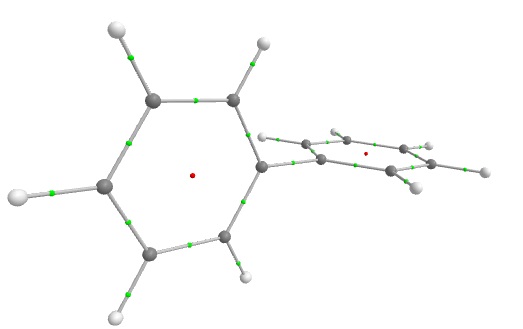 |
| --- | --- |
| 0^o^ | 90^o^ |

**Figure S10.** Molecular graphs and critical points of the biphenyl configuration at the planar and perpendicular energy barrier. The green points mark the bond critical points and the red points the ring critical points. Bond critical points appear between the *ortho* hydrogens in the planar conformation.

A relationship between the existence of a bond critical point (BCP) between two nuclei and their $V_{\text{X}}$value has been established[[2](#_ENREF_2)]. The relationship views pairs of nuclei as competitors for a BCP: the nuclei that are associated with the highest V_X_ value (in terms of absolute value) will be linked by a BCP. Figure S4 makes clear that $V_{\text{X}}^{H_{2}H'_{2}}$values are indeed the highest in the set of all $V_{\text{X}}^{HH}$values. Indeed, a bond critical point emerges between H_2_ and H_2_’ as soon as the central torsion angle falls below 28⁰. Figure S10 shows the complete situation in which a bond critical point also appears between H_6_ and H_6_’.

**References**

[1] J. Dillen Congested Molecules. Where is the Steric Repulsion? An Analysis of the Electron Density by the Method of Interacting Quantum Atoms, Int.J.Quant.Chem., **113** (2013) 2143-2153.

[2] A. Martín Pendás, E. Francisco, M.A. Blanco, C. Gatti, Bond Paths as Privileged Exchange Channels, Chem.Eur.J., **13** (2007) 9362-9371.
